# Polyunsaturated fatty acids and psychotic disorders: integrating mechanistic, genetic, and clinical evidence

**DOI:** 10.3389/fnut.2026.1929742

**Published:** 2026-09-16

**Authors:** Laura J. Westacott, Karolina Dec, Hanna Gruszczynska, Craig Joyce, Stephanie Mitchell, Jeremy Hall, Adrian J. Harwood

**Affiliations:** 1 Psychiatric Nutrigenomics Group, Neuroscience and Mental Health Innovation Institute, Cardiff University, Cardiff, United Kingdom; 2 Independent Researcher, Zürich, Switzerland; 3 Department of Psychiatry, Warneford Hospital, University of Oxford, Oxford, United Kingdom; 4 Department of Translational Genomics, Mental Health and Neuroscience Research Institute (MHeNs), Maastricht University, Maastricht, Netherlands

**Keywords:** schizophrenia, bipolar disorder, neurodevelopment, omega-3, omega-6, polyunsaturated fatty acids

## Abstract

Polyunsaturated fatty acids (PUFAs), including omega-3 (n-3) and omega-6 (n-6) essential fatty acids, play critical roles in brain development and function, serving as precursors to a wide range of bioactive lipid mediators involved in immune and inflammatory signaling. Emerging evidence implicates PUFA dysregulation in schizophrenia and bipolar disorder, severe psychiatric conditions that remain incompletely treated by current therapeutics. In this review, we first outline the biological roles of PUFAs relevant to neurodevelopment and psychiatric risk, with particular emphasis on their interactions with synaptic, immune, and stress-related risk pathways impinging on neurodevelopment. We synthesize preclinical evidence showing how altered PUFA availability influences neurobiological and behavioral phenotypes relevant to psychosis, alongside human observational, epidemiological, genomic and lipidomic studies that implicate disrupted PUFA metabolism in schizophrenia and bipolar disorder. Finally, we evaluate clinical trials of n-3 PUFA supplementation, considering both its therapeutic potential and current limitations. Together, we highlight PUFA metabolism as a mechanistic bridge linking neurodevelopment, immune function, and psychiatric risk, and as a potential target for future therapeutic strategies.

## 1 Introduction

There is growing interest in altered lipid metabolism as a contributor to psychiatric pathophysiology, driven by evidence of altered polyunsaturated fatty acid (PUFA) levels in individuals with schizophrenia and bipolar disorder, alongside genetic associations with variants in fatty acid metabolism genes. The involvement of phospholipid biology in psychotic disorders was first proposed in the 1990s with the development of the membrane phospholipid hypothesis of schizophrenia (1). This hypothesis posits that abnormalities in neuronal phospholipid metabolism contribute to disease pathophysiology through effects on membrane structure, signal transduction, and synaptic function. These processes are particularly critical during neurodevelopmental periods such as adolescence, a key window for synaptic maturation and pruning, as well as for maintaining normal brain function in adulthood. While initially focused on schizophrenia, emerging evidence suggests that similar alterations in phospholipid composition and PUFA metabolism are also relevant to bipolar disorder, despite historically receiving less attention. Although early support for this framework was based on observations of altered lipid profiles and membrane composition in patients, it has gained renewed relevance in light of recent genetic and lipidomic findings. Together, these data suggest that disruption of lipid metabolism may represent a shared mechanistic link between environmental exposures and biological risk pathways implicated across psychotic disorders.

In this review, we examine the evidence linking PUFA metabolism to schizophrenia and bipolar disorder, beginning with the diverse biological roles of PUFAs and their interactions with key pathways implicated in psychiatric risk, including neurodevelopment and immune signaling. We then synthesize preclinical evidence examining how manipulation of PUFA availability influences neurodevelopmental and behavioral phenotypes relevant to psychosis. Next, we review human observational studies of PUFA levels in blood, plasma, cerebrospinal fluid and brain tissue, alongside lipidomic analyses from cross-sectional and longitudinal cohorts. We also discuss genomic evidence, including genome-wide association studies and Mendelian randomisation analyses, that provides insight into potential causal links between PUFA metabolism and schizophrenia and bipolar disorder. Finally, we evaluate clinical trials of PUFA supplementation in patient and clinical high-risk populations, primarily as an adjunct to pharmacological treatment, before highlighting the key unanswered questions and future directions needed to translate these findings into clinical benefit.

## 2 Psychotic disorders

Psychotic disorders are a group of severe and highly disabling mental illnesses characterized by a loss of contact with reality. Core symptoms include hallucinations, delusions, and disorganized thought, speech, and behavior, commonly referred to as positive symptoms. In contrast, negative symptoms such as anhedonia, avolition, alogia, and blunted affect contribute substantially to functional impairment.

Diagnostic categories include schizophrenia, schizoaffective disorder, schizophreniform disorder, and brief psychotic disorder, as defined by DSM classifications. Although bipolar disorder is not classified as a primary psychotic disorder, it is included here due to the frequent presence of psychotic features during depressive and manic episodes (2). In addition, increasing evidence supports a dimensional rather than strictly categorical model of psychiatric illness, with substantial overlap across traditional diagnostic boundaries.

Schizophrenia and bipolar disorder show considerable convergence in clinical presentation and genetic architecture. A substantial shared genetic liability is well-established, with common risk loci converging on biological pathways involved in synaptic function, ion transport, and immune signaling, amongst others (3–6). Large-scale genomic studies further support this overlap. The Brainstorm Consortium (2018) reported strong genetic correlations across psychiatric disorders, with the highest overlap observed between schizophrenia and bipolar disorder (7). Similarly, polygenic risk score analyses demonstrate that schizophrenia genetic risk is associated with bipolar subtypes featuring psychosis, supporting a schizophrenia-bipolar spectrum (8). A recent cross-disorder analyses of approximately one million cases identified a shared genetic factor encompassing schizophrenia and bipolar disorder, enriched for genes expressed in excitatory neurons across developmental stages (9).

Schizophrenia and bipolar disorder each affect approximately 1% of the global population, although lifetime prevalence of psychotic symptoms is considerably higher (10). Both conditions are associated with substantial individual and societal burden. Life expectancy is reduced by approximately 15–20 years in schizophrenia (11) and 10–12 years in bipolar disorder (12). While suicide is a leading cause of mortality (11, 13), both disorders are also characterized by high rates of physical comorbidity. Individuals with schizophrenia show increased prevalence of major global mortality risk factors (14), and bipolar disorder is also associated with elevated mortality from cardiovascular, cerebrovascular, and respiratory diseases (13). Notably, metabolic abnormalities and dyslipidaemia are highly prevalent in both schizophrenia and bipolar disorder, and these extend beyond medication induced side-effects (15, 16). These combined factors contribute to the substantial individual and socioeconomic burden of psychotic disorders (17, 18).

Both disorders typically emerge during adolescence or early adulthood and follow either a chronic or relapsing course. Schizophrenia is generally characterized by a chronic trajectory, whereas bipolar disorder presents with episodic mood disturbances, including periods of mania and depression. Sex differences are also observed, with schizophrenia more prevalent and typically presenting earlier in males, although risk may increase in females during hormonal transitions such as perimenopause (19). Bipolar disorder shows broadly similar prevalence across sexes, though recent evidence suggests increasing prevalence in women and a potential association between perimenopause and first-onset mania (20).

Current pharmacological treatments, including antipsychotics and mood stabilizers, are effective for managing acute symptoms, particularly positive symptoms of psychosis. However, these treatments are often associated with significant adverse effects that limit adherence (21). Negative symptoms and cognitive deficits remain poorly addressed, and approximately 30% of patients exhibit treatment resistance (22). In bipolar disorder, mood stabilizers such as lithium and anticonvulsants are commonly used, but similarly show variable efficacy and tolerability (23). There remains a clear need to better understand underlying disease mechanisms and to develop more effective and targeted therapeutic strategies for both schizophrenia and bipolar disorder.

## 3 Dietary sources, absorption, and metabolism of polyunsaturated fatty acids

Polyunsaturated fatty acids are important components of mammalian diets and consist of the omega-3 (n-3) and omega-6 (n-6) families, which play critical roles in brain development and function (Figure 1). These classes can be further subdivided into short-chain (SC-PUFAs) and long-chain (LC-PUFAs). Short-chain PUFAs linoleic acid (LA; 18:2n-6) and alpha linolenic acid (ALA; 18:3n-3) are the dietary essential fatty acids and are primarily derived from plant sources. LA, the n-6 PUFA parent, is abundant in vegetable oils such as sunflower and soybean oils, whereas ALA, the n-3 PUFA parent, is found primarily in flaxseed, walnuts, chia seeds and rapeseed oil. The primary biological role of SC-PUFAs is to act as substrates (precursors) for the synthesis of LC-PUFAs. Through sequential processes of elongation and desaturation, LA and ALA are converted into the long-chain derivatives arachidonic acid (AA; 20:4n-6), eicosapentaenoic acid (EPA; 20:5n-3), and docosahexaenoic acid (DHA; 22:6n-3) (24). Preformed n-3 LC-PUFAs EPA and DHA are predominantly obtained from marine sources, such as oily fish and algal oils, whereas n-6 LC-PUFAs such as AA are typically found in poultry, meat and eggs.

**FIGURE 1:**
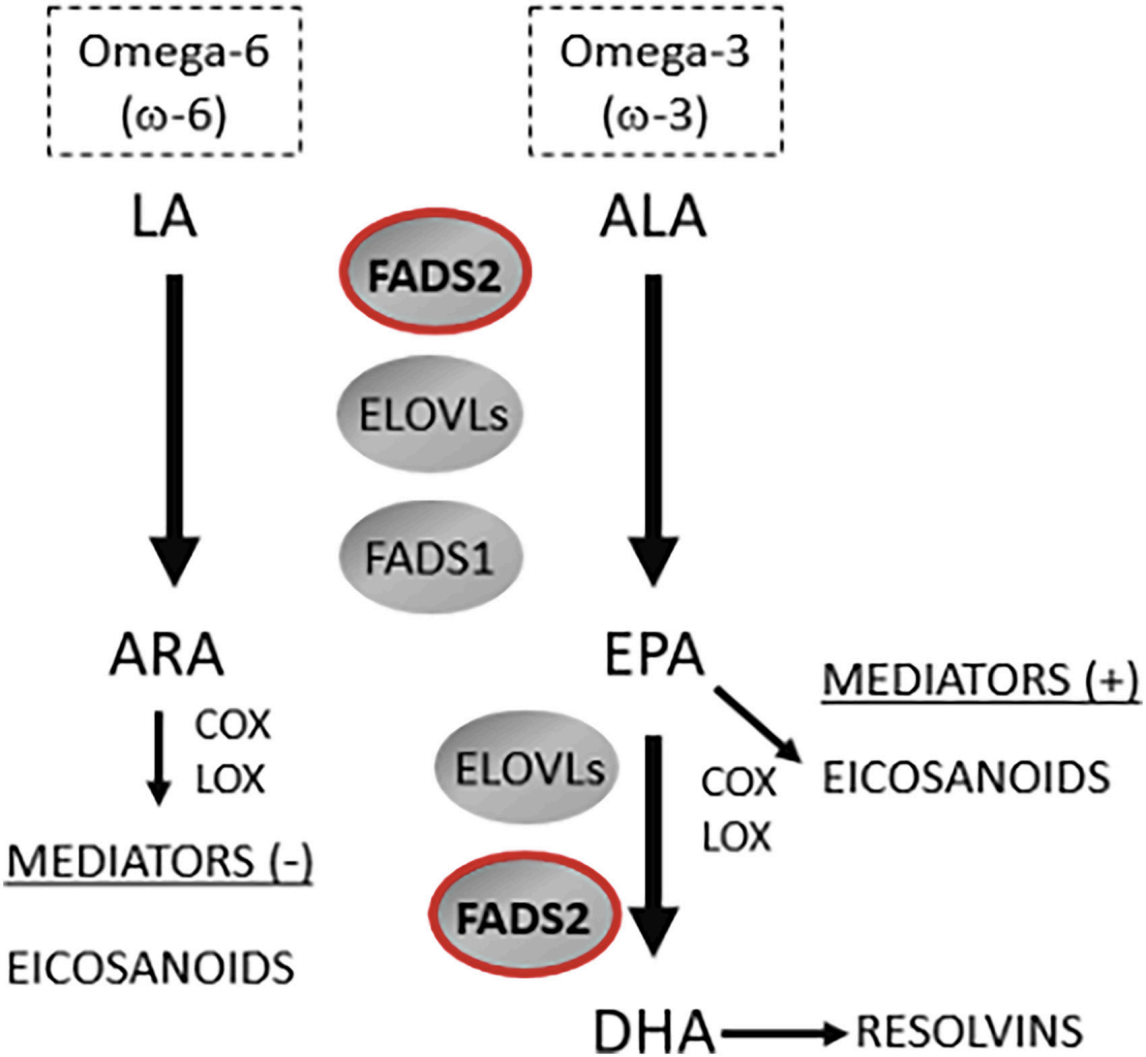
Overview of polyunsaturated fatty acid **(PUFA)** metabolic pathways. Dietary omega-6 (n-6) and omega-3 (n-3) essential fatty acids are metabolized through a shared series of desaturation and elongation reactions catalyzed by **Δ** 6-desaturase (FADS2), elongases (ELOVL family), and **Δ** 5-desaturase (FADS1). Linoleic acid (LA; n-6) and α -linolenic acid (ALA; n-3) compete for these enzymes, resulting in the production of arachidonic acid (ARA; n-6) and eicosapentaenoic acid (EPA; n-3), respectively. EPA is further elongated and desaturated to generate docosapentaenoic acid (DPA; n-3; not shown) and ultimately docosahexaenoic acid (DHA; n-3). Long-chain PUFAs may be esterified into membrane phospholipids, where they contribute to membrane structure and serve as reservoirs for bioactive lipids. Upon cellular activation, phospholipase-mediated release of these fatty acids enables their conversion into lipid mediators via cyclooxygenase **(COX)** and lipoxygenase **(LOX)** pathways. These mediators, including eicosanoids derived primarily from AA and resolvins derived from EPA and DHA, exert diverse and often opposing effects on inflammation and its resolution.

During conversion of SC to LC PUFAs, c*is* double bonds are introduced at specific positions in a fatty acid chain by fatty acid desaturase (FADS) enzymes. Δ-5 desaturase (D5D; FADS1), Δ-6 desaturase (D6D; FADS2), elongase 2 (Elovl2), and elongase 5 (Elovl5) are the enzymes involved in these reactions. Importantly, FADS2 is the primary rate-limiting factor in the biosynthesis of LC-PUFAs in mammals since it catalyzes the first step in the biosynthesis of LC-PUFAs from 18-carbon PUFAs (25). n-3 and n-6 lipid species compete for the same rate-limiting enzymes (26) and exert distinct physiological functions in the human body.

The conversion of SC- to LC-PUFAs in humans is limited by low conversion efficiency; however, studies indicate a low rate of conversion between ALA to EPA of approximately 8% and an even lower rate of ALA to DHA conversion, between 0.4% and 9% (27, 28). The conversion rates vary considerably between tissues and are modulated by several genetic, physiological and dietary factors. Oestrogen status, for instance, affects PUFA metabolism with women of reproductive age demonstrating substantially greater conversion ALA to DHA than men (29–31). *FADS* haplotypes also significantly impact LC-PUFA conversion, with genotype accounting for 28% of variation in blood AA levels (32, 33). Furthermore, conversion efficiency is highly dependent on dietary fatty acid intake, particularly EPA status and dietary ratio of n-6 and n-3 SC-PUFA’s (26, 34) meaning that dietary consumption of preformed LC-PUFAs is generally considered necessary to achieve adequate LC-PUFA concentrations (27).

In Western populations, dietary availability of n-6 short-chain PUFAs increased substantially over the 20th century, while intake of n-3 short- and long-chain PUFAs declined, with average oily fish consumption in the UK estimated to be ∼40% below recommended levels (33, 34). Furthermore, this pattern is no longer confined to Western societies, as dietary “westernization” is increasingly observed globally (35, 36). Consequently, many contemporary dietary patterns globally are characterized by relatively high intakes of n-6 PUFAs, particularly linoleic acid, driven in part by widespread use of vegetable oils such as soybean oil in ultra-processed foods (35), alongside greater consumption of red meat and dairy products, compared with the dietary pattern on which humans evolved. In contrast, dietary patterns rich in fish, legumes, fruit, and vegetables, such as the Mediterranean diet, are higher in n-3 PUFAs and are associated with a range of beneficial health effects (37, 38).

A recent cross-continental analysis using dried blood spots for determination of n-6 and n-3 levels confirmed a widespread inadequacy of n-3 levels and imbalanced n-6:n-3 ratios (30). Lowest n-3 levels were found in North America, and highest in Asia, Europe and Oceania. Such dietary shifts have resulted in a large increase in the n-6:n-3 ratio from an estimated 1:1 during human evolution to 20:1 in present day, though ratios may be as high as 50:1 in the United States (39–41). At present, a ratio of 1:1 to 5:1 is considered optimal for human health (24, 42). Another frequently used measure is the omega-3 Index, which reflects the sum of EPA plus DHA as a percentage of total measured fatty acids in erythrocyte cell membranes. An optimal omega-3 index is 8%–11% (43) and is seen in countries with high-seafood intake such as Japan, Alaska and South Korea whereas low indexes of ∼4% were found in the US, Canada and some European countries (44). The omega-3 index has been found to never fall below 2% in large studies, suggesting this is the absolute minimum level of EPA and DHA required (45). Imbalances in n-6:n-3 ratio have been associated with a range of morbidities, contributing to atherosclerosis, obesity, cardiovascular disease, cancers, metabolic disorders and neuropsychiatric conditions (30, 46).

## 4 Genomics of fatty acid biosynthesis

The *FADS* gene cluster plays a central role in PUFA biosynthesis and includes fatty acid desaturase 1 (*FADS1*), which encodes the D5D enzyme, and fatty acid desaturase 2 (*FADS2*) which encodes D6D. These genes show 75% sequence similarity to one another (47). A third *FADS* gene, fatty acid desaturase 3 (*FADS3*), was identified in 2000 and shares 62% and 70% sequence similarity with *FADS1* and *FADS2*, respectively (25). All three genes sit within a 92 kb cluster on 11.q12-q13.1 (47). Other members of the PUFA metabolism pathway include Elovl2 which is encoded by *ELOVL2*, and Elovl5, encoded by *ELOVL5* (24). As discussed, D6D (FADS2) is the primary rate-limiting enzyme in mammalian LC-PUFAs biosynthesis, since it catalyzes the first step in the conversion of 18-carbon SC-PUFAs to LC-PUFAs. Of note, the function of *FADS3* remained elusive as it was thought to lack enzymatic capability of other desaturases (48). More recently, it has been demonstrated that *FADS3* is a bona fide desaturase required for conversion of sphingolipids (49). While it is still not thought to participate in PUFA biosynthesis, its expression has been found to increase in response to downregulation of *FADS1* and *FADS2* (50). The *FADS* cluster genes are expressed widely across a human tissue, including in the brain, and common *FADS* cluster variants are known to impact transcription in a variety of tissues (51).

Polymorphisms in *FADS* and *ELOVL* genes have been reported in multiple ancestries and shown to strongly regulate levels of LC-PUFA biosynthesis, regardless of dietary intake (33, 52, 53). Single-nucleotide polymorphisms (SNPs) within these genes are associated with multiple phenotypes at a genome-wide significant level, including lipid metabolism, glucose metabolism, and traits such as total cholesterol and low-density-lipoprotein (52). Furthermore, genomic studies have implicated the *FADS* gene cluster in risk for both bipolar disorder and schizophrenia (5, 54–57); these associations are discussed in greater depth in Section 9. This evidence suggests the possibility that low dietary intake of long-chain n-3 PUFAs, or an imbalanced n-6:n-3 ratio, may exacerbate functional deficiencies in individuals carrying SNPs that reduce desaturase activity, potentially increasing risk for psychiatric disorders and other morbidities (26) although direct experimental evidence to support this hypothesis is currently lacking.

Therefore, interindividual differences in PUFA status are determined not only by dietary intake but also by genetic variation in the enzymes responsible for fatty acid desaturation and elongation. Variants within the FADS and ELOVL gene families can substantially influence the efficiency with which precursor fatty acids are converted into biologically active long-chain PUFAs. Consequently, understanding the physiological functions of PUFAs in the brain requires consideration of both nutritional and genetic influences on PUFA metabolism. The biological significance of these pathways is discussed in the following section.

## 5 Polyunsaturated fatty acids and their biological roles in the developing and adult brain

Polyunsaturated fatty acids are essential components of neuronal membranes and play critical roles in brain development, synaptic function, and inflammatory regulation. The primary forms of LC-PUFAs in the brain are AA and DHA, which are primarily present as components of membrane phospholipids. Together, these lipids account for approximately ∼20% of total brain lipids (58), with DHA comprising around 60% of brain PUFAs, whereas EPA represents only ∼0.1% of brain lipids (59). Although monounsaturated and saturated fatty acids are synthesized endogenously by the brain, evidence suggests that the brain relies on a constant influx of PUFAs from the bloodstream (60). Preclinical radiolabelling studies demonstrated rapid turnover of both AA and DHA in the brain, with human PET studies estimating daily metabolic consumption of approximately 4.6 mg of DHA and 17.8 mg of AA, representing their incorporation and turnover in brain membrane phospholipids rather than their use as an energy source (61). Despite this ongoing turnover, DHA has a prolonged brain half-life of approximately 2.5 years, reflecting its substantial retention in brain membranes (62). Dietary fatty acid composition influences brain PUFA status; elevated dietary n-6 intake or n-3 deficiency reduces brain DHA and increases AA concentrations in experimental models (58, 63).

Notably, the developing human brain accumulates fatty acids throughout fetal development, with a marked acceleration during the final weeks of gestation, resulting in substantial stores of LA, AA, and DHA in full term infants (64) highlighting their importance for neurodevelopment and normal brain maturation (65). DHA is particularly enriched within synaptic membranes and the gray matter of the brain, where it contributes to membrane fluidity, receptor function, ion channel activity, synaptic plasticity, and signal transduction (60, 66). AA is also highly concentrated in neuronal phospholipids and plays important roles in intracellular signaling pathways and neurotransmitter release.

In addition to their structural functions, alterations in membrane PUFA composition affect membrane fluidity and the organization of lipid rafts, thereby influencing the function of membrane-bound receptors, transporters, and intracellular signaling proteins (67, 68). Experimental studies demonstrate that n-3 LC-PUFAs can modulate dopaminergic, serotonergic, glutamatergic, and endocannabinoid neurotransmission, systems that have been strongly implicated in the pathophysiology of psychotic disorders (60, 69). Furthermore, DHA is an important constituent of myelin membranes and may contribute to oligodendrocyte function and white matter (70, 71).

Polyunsaturated fatty acids stored in an esterified form in membrane phospholipids can be released to generate bioactive lipid mediators. LC-PUFAs, including EPA, DHA, and AA, are released from cell membranes by phospholipase A_2_ (PLA_2_) and metabolized by cyclooxygenases (COXs), lipoxygenases (LOXs), and cytochrome P450 enzymes to produce signaling molecules known as oxylipins. Active LC-PUFA metabolites include eicosanoids and docosanoids (26). Eicosanoids, such as prostaglandins, thromboxanes, and leukotrienes, derived primarily from AA, EPA, and dihomo-γ-linolenic acid (DGLA), are potent regulators of inflammation and homeostasis (72, 73). These mediators can exert divergent effects, with AA-derived eicosanoids generally promoting pro-inflammatory responses, whereas EPA-derived species are less inflammatory or anti-inflammatory. In contrast, docosanoids, including resolvins, protectins, maresins, and neuroprotectin D1, actively promote the resolution of inflammation. These compounds are collectively referred to as specialized pro-resolving mediators (SPMs), which have been implicated in the regulation of neuroinflammation, although their reliable detection, physiological concentrations and relevance *in vivo* remain subject to ongoing debate (74–76). Unlike classical anti-inflammatory agents, SPMs actively coordinate the termination of inflammatory responses and promote tissue repair, restoration of homeostasis, and clearance of cellular debris (77). Neuroprotectin D1, a DHA-derived mediator enriched in neural tissue, has demonstrated neuroprotective, anti-apoptotic, and anti-inflammatory effects in experimental models, suggesting a potential role for n-3 LC-PUFAs in maintaining central nervous system homeostasis (77, 78).

The balance between n-6 and n-3 fatty acids is a key determinant of immune homeostasis, influencing the equilibrium between pro-inflammatory and anti-inflammatory processes via the production of downstream lipid mediators (46, 79). In individuals exposed to western dietary patterns, AA predominates within cell membrane phospholipids (∼20%), compared to substantially lower levels of EPA and DHA (80). Increasing intake of pre-formed n-3 LC-PUFAs, such as through fish oil supplementation, enriches membrane EPA and DHA at the expense of AA and is associated with reduced production of pro-inflammatory eicosanoids by immune cells (72, 81). Together, these findings highlight dietary PUFA composition as a key determinant of lipid mediator profiles, with the potential to shift inflammatory tone and downstream immune signaling.

Of note, the n-3 SC-PUFA ALA is primarily considered a precursor to the n-3 LC-PUFAs (27); however, ALA appears to play distinct roles in several areas of human health, including supporting cardiovascular function and brain and nerve development (82). Patterns of high dietary intake of ALA are associated with neurogenic and neuroprotective effects, including reduced risk of neurological disease, altered neuroinflammatory processes, and reduced cognitive impairment in cohort studies (83, 84). Mechanistic evidence for specific biological activity of ALA is growing, particularly in neurobiology, where preclinical studies suggest a high affinity for ALA-to-EPA conversion in cerebral tissue (85). ALA may modulate neuroinflammatory pathways, neurogenesis, and neuronal survival independently of LC-PUFAs via bioactive oxylipin mediators (84, 86) and has also been shown to influence nuclear factor kappa-light-chain-enhancer of activated B cells (NF-κB) and brain-derived neurotrophic factor (BDNF) mRNA expression (28, 87–90).

Emerging evidence further suggests that the balance between dietary LA and ALA may influence neurodevelopment. Using human induced pluripotent stem cell (iPSC) derived neuronal cultures, Dec et al. (91) demonstrated that a high LA:ALA ratio adversely affected early neuronal differentiation and reduced the electrophysiological activity of glutamatergic neurons, suggesting that excessive n-6 relative to n-3 SC-PUFA exposure may impair early neurodevelopmental processes. Mechanistically, these effects may reflect altered competition between LA and ALA for desaturation and elongation enzymes, resulting in changes in downstream LC-PUFA synthesis and metabolite production. Alternatively, or additionally, they may arise from direct effects of LA-derived oxylipins and other bioactive lipid mediators on neuronal differentiation and function. These findings are particularly relevant given that Western dietary patterns are characterized by relatively high LA:ALA ratios, which may influence both LC-PUFA availability and the balance of downstream lipid signaling pathways. Preclinical *in vivo* evidence also indicates that n-3:n-6 PUFA ratios during gestation can influence offspring neurodevelopment. In mice, maternal diets enriched in either n-3 or n-6 PUFAs altered the development of neurological reflexes relative to control animals (92, 93). Although accelerated development of neurological reflexes may confer an adaptive advantage under certain environmental conditions (93) both deficiency and excess of n-3 and n-6 PUFAs, respectively, were associated with adverse developmental outcomes. These findings suggest that an appropriate balance between n-3 and n-6 PUFAs, rather than an absolute enrichment of either fatty acid class, may be important for optimal neurodevelopment.

The central roles of PUFAs in membrane architecture, neurotransmission, inflammatory regulation, myelination, and neurodevelopment underscore their importance in maintaining normal brain structure and function. Consequently, disturbances in PUFA availability or metabolism may have far-reaching effects on neural processes that are increasingly implicated in psychiatric disorders.

## 6 Mechanistic links between polyunsaturated fatty acids and psychiatric risk pathways

The diverse biological functions of PUFAs position them to influence several pathways implicated in schizophrenia and bipolar disorder, including neurodevelopment, synaptic function, neurotransmission and inflammatory signaling. These interactions provide a potential mechanistic link between genetic variation in PUFA metabolism, environmental exposures, including dietary PUFA intake, and psychiatric risk. This section therefore examines evidence that PUFAs modulate established risk pathways involved in the pathophysiology of psychotic disorders.

Dysregulated inflammation is increasingly recognized as a putative mechanism in schizophrenia and bipolar disorder, with evidence of altered immune profiles and genetic risk loci enriched within immune-related pathways (94–96). In addition, prenatal and childhood exposure to infection is a well-established environmental risk factor for schizophrenia [reviewed in Cheslack-Postava and Brown (97)]. Given their immunomodulatory properties, PUFAs may influence susceptibility to these immune-associated risk factors. In animal models, n-3 PUFA supplementation enriches brain tissue with anti-inflammatory, pro-resolving oxylipins while reducing pro-inflammatory n-6-derived species (98). Maternal immune activation (MIA) is a well-established paradigm that mimics risk for schizophrenia associated with prenatal exposure to infection (99). In this context, maternal n-3 deficiency exacerbates offspring behavioral and neurobiological abnormalities, including persistent alterations in inflammatory signaling (100), whereas n-3 supplementation attenuates neuroinflammatory and behavioral changes in both rat and mouse MIA models (101–103). Similarly, n-3 deficiency promotes a pro-inflammatory oxylipin profile in lipopolysaccharide (LPS) models of immune challenge, whereas DHA and its downstream lipid mediators suppress microglial activation through reduced IL-6 secretion and inhibition of NF-κB, IL-1β, and TNF-α signaling (104, 105). Together, these findings suggest that PUFAs may modulate immune pathways implicated in psychiatric risk by promoting anti-inflammatory signaling in response to immune activation.

Immune pathways also contribute to neurodevelopmental processes relevant to psychosis, including synaptic pruning. This process, which refines neural circuits throughout development and into early adulthood, is mediated in part by innate immune mechanisms such as complement-dependent tagging of synapses and microglial phagocytosis or trogocytosis (106, 107). Aberrant complement-mediated pruning is a leading candidate disease mechanism accounting for the synapse loss observed in schizophrenia [reviewed in Westacott and Wilkinson (95)], and structural variation in the complement C4A gene confers elevated risk for the disorder (108).

Converging evidence indicates that n-3 PUFAs influence this process through effects on microglial phenotype and function. Gestational n-3 enrichment alters offspring microglial lipid composition, increasing DHA and reducing n-6 docosapentaenoic acid (DPA) levels (98, 109), whereas n-3 deficiency promotes microglial activation, enhances synaptic phagocytosis, and upregulates innate immune and complement-related genes. These alterations are accompanied by reduced dendritic spine density and impaired recognition memory, consistent with excessive synaptic pruning (109). Notably, the complement cascade emerged as the most significantly altered pathway following n-3 supplementation in individuals at ultra-high risk for psychosis (110). In this study, changes in complement proteins mediated the beneficial effects of n-3 supplementation on symptom severity, providing translational support for interactions between PUFA biology and complement-dependent neurodevelopmental processes. These findings are particularly noteworthy given that the ultra-high-risk period often coincides with a critical developmental window during adolescence, during which higher-order brain regions implicated in schizophrenia, including the prefrontal cortex, are thought to undergo ongoing synaptic refinement. Consequently, these findings raise the possibility that PUFAs may represent a promising avenue through which immune-mediated synaptic pruning processes can be modulated in at-risk individuals.

The effects of PUFAs on synaptic pruning represent only one aspect of their broader role in neurodevelopment. Environmental risk factors for schizophrenia are concentrated during gestation and early childhood, while genetic risk factors converge on pathways involved in early neurodevelopment, suggesting disruption of these processes contribute to disease risk (111). Consistent with this, n-3 PUFAs influence neurogenesis (112, 113), cortical and hippocampal progenitor cell migration (114), neurite outgrowth (115), and synaptogenesis (112, 116). In human iPSC models of forebrain development, a 5:1 LA:ALA ratio, representing an n-6:n-3 PUFA imbalance, disrupted neuronal progenitor proliferation and differentiation through the downregulation of *FOXG1*, a key regulator of neurogenesis and maturation (91). Therefore, evidence supports a role for PUFAs in coordinating early neurodevelopmental processes that are implicated in psychosis risk.

Polyunsaturated fatty acids may also contribute to white matter development and myelination, processes increasingly implicated in schizophrenia and bipolar disorder. Neuroimaging studies have identified widespread white matter abnormalities and altered connectivity in bipolar disorder and schizophrenia (117, 118). PUFAs may influence these processes through effects on microglia, which play key roles in lipid processing, myelin remodeling, and oligodendrocyte maintenance (119, 120). In animal models, maternal n-3 deficiency reduces white matter-associated microglial density and microglia–myelin interactions within the developing corpus callosum (121), a region in which white matter abnormalities have been found in bipolar disorder (118). n-3 deficiency further impairs oligodendrocyte maturation, resulting in thinner myelin sheaths and disrupted white matter organization (100). Although some of these structural alterations appear transient, they are associated with persistent behavioral abnormalities in adulthood, including impaired learning, repetitive behaviors, and anhedonia-like phenotypes (100). Complementing these experimental findings, Peters et al. (71) demonstrated that *FADS* haplotypes associated with lower circulating levels of LC-PUFAs are correlated with altered patterns of age-related white matter differences in humans from childhood to adulthood. Moreover, *FADS1/2* expression in oligodendrocytes is associated with maintaining cognitive function, suggesting that variation in PUFA metabolism within myelinating cells may have functional consequences in humans (122). These findings support a role for PUFAs in white matter maturation and myelin integrity, providing another potential mechanism through which altered PUFA metabolism may contribute to psychiatric vulnerability.

Genomic studies identify the synapse as a central driver of schizophrenia risk, with multiple susceptibility loci converging on synaptic signaling pathways (123–126). Beyond their role in synaptic pruning, PUFAs directly influence synaptogenesis, neuronal activity, and synaptic plasticity. Across *in vitro* and *in vivo* models, in multiple species, n-3 deficiency impairs synaptogenesis, neurotransmitter release, action potential firing, and long-term potentiation (91, 112, 127–130), whereas DHA supplementation restores function (112, 116, 127) Through conversion into bioactive lipid mediators, including AA derived endocannabinoids, PUFAs also regulate postsynaptic excitability and long-term synaptic plasticity (77, 127). Intriguingly, n-3 PUFAs may act on several pathways implicated in the therapeutic effects of antipsychotic medications, including dopaminergic and glutamatergic signaling (131). Given the central role of dopamine and NMDA receptor dysfunction in current models of schizophrenia pathophysiology (132), these observations raise the possibility that PUFAs could complement existing pharmacological approaches, a hypothesis that has prompted interest in their use as adjunctive treatments (131). Consistent with this, n-3 deficiency produces mesocortical dopamine hypoactivity alongside mesolimbic hyperactivity in rats, mirroring patterns seen in patients (133). The interaction between PUFAs and neuronal signaling relevant to schizophrenia has been investigated in an *NR1* (Grin1/GluN1) knockdown mouse model of NMDA receptor hypofunction. These mice exhibit schizophrenia-relevant behavioral phenotypes and, under control diets, altered brain PUFA composition, including increased n-6 LA, despite no corresponding changes in serum, suggesting that NMDA hypofunction itself alters brain PUFA metabolism. Dietary n-3 deficiency further altered brain lipid composition, increased n-6 accumulation, and exacerbated mortality, indicating greater vulnerability to PUFA deficiency in the context of NMDA hypofunction (134). Notably, n-3 supplementation did not rescue behavioral deficits, suggesting that increased DHA cannot compensate for impaired NMDA receptor signaling. Together, these findings indicate that NMDA hypofunction may disrupt brain PUFA homeostasis and increase susceptibility to dietary PUFA imbalance. In human iPSC forebrain glutamatergic neurons, a 5:1 ratio of n-6:n-3 SC-PUFAs induced expression of GABAergic receptors and abnormal electrophysiological activity (91). Therefore, PUFA-dependent regulation of synaptic and neurotransmitter systems may interact with functional neurobiological alterations underlying psychosis risk.

n-3 PUFAs regulate neurotransmission through both membrane-dependent and direct mechanisms. By incorporating into neuronal membranes, they alter membrane biophysical properties and fluidity, while they can also directly modulate the function of membrane proteins. For example, PUFAs regulate multiple ion channels, including voltage-gated calcium channels (VGCCs; Ca_v_), by modifying channel gating and voltage sensitivity (135). Variants in *CACNA1C*, encoding the Ca_v_1.2 channel subunit, represent one of the strongest shared genetic risk factors for bipolar disorder and schizophrenia, implicating disrupted calcium signaling in psychotic disorders [reviewed in Moon et al. (136)]. Although direct links between VGCCs and circulating PUFA levels remain unclear, mechanistic evidence indicates potential convergence between these pathways. Firstly, intracellular Ca^2+^ activates cytosolic phospholipase A_2_ (cPLA_2_), which cleaves membrane phospholipids to release LC-PUFAs, particularly AA, which are subsequently metabolized into eicosanoids via COX/LOX pathways (137, 138). Altered cPLA_2_ activity has been reported in post-mortem brain tissue from individuals with schizophrenia, but not bipolar disorder, suggesting disorder-specific alterations in phospholipid signaling (139). cPLA_2_ has also been proposed to influence inflammatory signaling and, consequently, monoaminergic neurotransmission [reviewed in Nakamura (140)]. Thus, Ca^2+^-dependent cPLA_2_ activation provides a plausible link between altered calcium signaling and PUFA metabolism; however, this interaction has not, to our knowledge, been directly investigated in psychosis or bipolar disorder. Given independent evidence for abnormalities in both calcium signaling and cPLA_2_ activity, particularly in schizophrenia, this represents an important area for future investigation. In addition, PUFAs modulate Ca_v_ channels by reducing maximal conductance and shifting both activation and steady-state inactivation to more negative potentials, with a larger shift in inactivation, collectively reducing neuronal excitability (135, 141, 142). These actions appear to be independent of PUFAs impact on membrane fluidity and instead point to a direct binding action between PUFAs and Ca_v_ channels (143).

Other mechanistic links between PUFAs and VGCCs may involve caveolins, membrane scaffolding proteins that form caveolae, lipid-rich invaginations of the plasma membrane that organize signaling complexes. Cardiac Ca_v_1.2 channels localize to caveolae in association with caveolin-1/2/3, linking caveolar structure to calcium signaling (144, 145), although it is unknown whether this occurs in the brain. In cardiomyocytes and endothelium, n-3 and n-6 PUFAs exert opposing effects on caveola formation, with n-6 promoting and n-3 disrupting caveolar assembly (146), suggesting that PUFA-dependent changes in membrane fluidity may conceivably alter colocalised Ca_*V*_ channel cell surface expression and downstream calcium signaling. Evidence remains inconclusive as to whether this mechanism is present in the brain however. Therefore, through effects on Ca_v_ channel kinetics and cell surface expression, PUFA modulation of channel function may influence neuropsychiatric risk and may also theoretically modify the pharmacological effects of VGCC blockers, although this remains an unexplored area.

Finally, PUFAs may influence psychiatric risk through stress-response systems. Early life stress is a well-established risk factor for schizophrenia and bipolar disorder [reviewed in Robinson and Bergen (147)]. In animal models, n-3 deficiency disrupts HPA-axis regulation, increasing corticosterone responses to stress, altering glucocorticoid receptor signaling and prefrontal pyramidal neuron morphology; these effects can be reversed by n-3 supplementation (148–150). Early developmental n-3 deficiency may be particularly important, with maternal deficiency during gestation and weaning reducing hypothalamic DHA and enhancing stress-induced corticosterone responses in offspring (150). More recently, chronic n-3 deficiency from gestation to adulthood increased hypothalamic *Avp* and *Avpr1b* expression, suggesting altered vasopressin signaling as a potential mechanism linking n-3 deficiency to anxiety- and mood-related phenotypes (149). Conversely, n-3 PUFAs prevent glucocorticoid- and pro-inflammatory cytokine–induced anti-neurogenic effects in human hippocampal neural progenitor cells (151, 152), and DHA protects against glucocorticoid-induced neuronal death in cortical neurons *in vitro* (153). In humans, n-3 supplementation reduces stress-induced cortisol responses (154) and may normalize stress reactivity in chronically stressed individuals (155). Notably, chronic psychological distress is associated with increased n-6:n-3 ratios in both humans and non-human primates (156, 157), suggesting that PUFA metabolism may be altered under sustained stress conditions. These findings suggest that n-3 PUFAs may buffer against stress-induced neurobiological changes and associated psychiatric risk.

In summary, converging mechanistic evidence suggests that PUFA availability may influence vulnerability to psychiatric disorders through effects on interconnected neurodevelopmental processes, including synaptic refinement, neurotransmission, stress responsivity, and myelination. Rather than acting through a single pathway, PUFAs likely modulate multiple interacting systems that collectively shape brain development and disease risk. These effects are likely moderated by genetic variation in PUFA metabolism (e.g., *FADS* genotype) and by dietary factors. However, while these findings provide biological plausibility, they do not establish causality for psychosis-related phenotypes.

## 7 Preclinical animal studies of polyunsaturated fatty acid availability and psychosis-relevant phenotypes

The evidence outlined above suggests that PUFAs may mechanistically interact with multiple neuropsychiatric risk pathways. Experimental animal models provide an important opportunity to manipulate PUFA availability under controlled conditions, enabling stronger causal inference regarding effects on neurobiological and behavioral phenotypes relevant to psychosis. Preclinical animal studies have shown that dietary PUFA manipulation, particularly during the perinatal period, can shape neurodevelopmental trajectories and alter psychosis-relevant behavioral and neurobiological phenotypes. Rodent models manipulating n-3 and n-6 PUFA intake, most commonly via maternal diet during gestation and lactation, have been widely used to model Western dietary patterns and probe mechanisms relevant to neuropsychiatric disorders. In this literature, brain tissue and cellular PUFA composition has been shown to change considerably in response to neonatal dietary n-3 deficiency or supplementation (109, 158, 159). In addition, animal models manipulating *FADS1/2* provide further insight into how impaired endogenous PUFA synthesis influences behavioral phenotypes (160).

Offspring exposed to low n-3 PUFA availability during gestation exhibit increased stress reactivity, anxiety-like behaviors (148, 149, 161) and altered mood-related phenotypes (128, 149). In support, n-3 supplementation promoted resiliency in mice exposed to chronic social defeat stress (162). Diets deficient in n-3 PUFAs, including the n-3 SC-PUFA ALA, produce deficits in pre-pulse inhibition (PPI) of the acoustic startle response (163), a measure of sensorimotor gating that is consistently impaired in schizophrenia and bipolar disorder (164, 165). Fedorova et al. reported that animals receiving diets containing the n-3 LC-PUFAs EPA and DHA during gestation and early adulthood exhibited improved PPI compared with those receiving n-3 ALA-enriched diets, suggesting that preformed LC-PUFAs may be particularly important for normal sensorimotor gating (163). Furthermore, across multiple studies, n-3 deficient animals exhibit impairments in learning and cognitive flexibility (100, 109), including increased perseverative behavior (166). Deficits in cognition, decision-making, and increased perseverative errors are well-established features of schizophrenia and related psychotic disorders (167).

In the established “two-hit” model of schizophrenia, consisting of prenatal exposure to the viral mimic polyI:C and subsequent peripubertal stress (mimicking dual risk mechanisms arising from prenatal infection and childhood stress), n-3 supplementation prevented deficits in PPI, social interaction and working memory (101). In addition, chronic n-3 PUFA deficiency has been reported to potentiate the development of amphetamine-induced hyperlocomotion, a behavioral phenotype thought to model mania-like behavior, which is subsequently reversed by lithium chloride treatment (168). Together, these findings indicate that inadequate n-3 PUFA availability during brain development leads to adult behavioral phenotypes relevant to both schizophrenia and bipolar disorder.

Double heterozygous knockout mice lacking both *FADS1* and *FADS2* alleles have provided mechanistic insights into how common variation within this gene cluster may contribute to bipolar disorder pathogenesis. Yamamoto et al. (160) observed cyclic bouts of hypoactivity and hyperactivity accompanied by mood-related behavioral alterations, resembling key features of bipolar disorder. Interestingly, hyperactive episodes were more pronounced in male *FADS1/2* heterozygous mice than in females, whereas females displayed more chronic periods of hypoactivity. In addition, increased levels of n-6 PUFA metabolites were detected in the plasma of *FADS1/2* mutant mice despite no differences in brain DHA content. Dietary supplementation with DHA and EPA rescued both locomotor and depressive-like behavioral phenotypes in mutant mice of both sexes, whereas EPA supplementation alone was ineffective. Importantly, brain-specific conditional *FADS1/2* heterozygous deficiency did not produce comparable behavioral phenotypes, suggesting that altered *FADS1/2* activity in peripheral tissues contributes to the observed behavioral abnormalities (16, 160).

Several limitations of the current literature should be considered. Preclinical animal studies vary substantially in dietary formulations, timing of exposure, and the specific PUFAs manipulated, complicating direct comparisons across studies. Standard rodent diet composition represents a further source of heterogeneity, as chow typically contains a relatively high n-6:n-3 ratio, potentially influencing baseline phenotypes and modifying responses to experimental interventions. In addition, though manipulation of precursor levels during gestation has reliably produced changes in brain PUFA composition (98, 109, 128) the extent to which dietary DHA supplementation specifically can meaningfully alter brain DHA levels remains debated, with evidence suggesting it may be highly specific depending on formulation (159, 169). Brain PUFA uptake also differs between commonly used laboratory mouse strains (170). However, findings from *FADS1/2* manipulation suggest that behavioral and psychiatric-relevant phenotypes may arise independently of substantial alterations in brain PUFA composition, indicating that downstream effects on lipid signaling, metabolism, or peripheral physiology may be equally important determinants of risk (160). Despite these limitations, the overall evidence is broadly consistent across studies in demonstrating that n-3 PUFA deficiency produces psychiatric-relevant outcomes in preclinical animal models. Human observational studies represent an important next step in evaluating the relationship between PUFAs and psychotic disorders across different stages of illness and clinical risk.

## 8 Human observational evidence linking PUFAs with psychotic disorders

A substantial body of observational, cross-sectional, and longitudinal research has investigated the relationship between PUFAs and psychotic disorders, with a growing literature also emerging in bipolar disorder. Human studies have primarily quantified n-3 and n-6 PUFA status using biomarkers measured in red blood cell (erythrocyte) membranes, plasma, and, less commonly, cerebrospinal fluid (CSF) and post-mortem brain tissue. Cross-sectional studies have been instrumental in identifying associations between PUFA profiles and psychiatric symptoms, disease status, and clinical outcomes. In parallel, longitudinal and epidemiological studies have leveraged repeated sampling to examine how PUFA status relates to the subsequent development, progression, or severity of psychiatric symptoms. Together, these approaches have provided important insights into the potential role of PUFAs in the pathophysiology of psychosis and bipolar disorder. A summary of the key human studies examining PUFA status in psychosis-spectrum disorders is provided in Table 1.

**TABLE 1:** Summary of polyunsaturated fatty acid (PUFA) composition findings in psychosis from key human observational studies and meta-analyses included in this review.

References	Evidence type	Disorder and sample size	Age group (years)	PUFA status	Conclusions
Meta-analyses					
Van der Kemp et al. (176)	Meta-analysis of 14 studies	429 SCZ (Med, NM); 444 NC	Late teens/early 20s to ∼50	AA↓, DHA↓, DPA↓ in Med and NM; LA↓, DGLA↓, EPA↓ and DTA↓ in Med (typical antipsychotics) DHA↓ in Med (atypical psychotics)	ERY PUFAs↓ in SCZ, present even in NM.
Hoen et al. (180)	Meta-analysis of 18 studies from 22 cohorts	642 (169 NM) SCZ; 574 NC	First-episode young adults to chronic older adults: 17–75	DPA↓, DHA↓ in Med and NM LA↓ in Med only AA↓ in NM	ERY DPA↓, DHA↓ and AA↓ may be associated with SCZ independent of the effects of psychotics.
McNamara and Welge (181)	Meta-analysis of 6 CC studies	118 BD; 147 NC	Children, adolescents to middle-aged adults: 9–65	Significant DHA↓ and a trend for EPA↓. No changes in LA/AA.	ERY DHA↓ in BD, implicating n-3 deficiency in its pathophysiology.
Gao et al. (188)	Umbrella review of 95 meta-analyses from 29 systematic reviews	Multiple studies comprising 1000 s of participants across disorders, including SCZ and BD	Children to older adults	↓Circulating n-3 PUFAs	Overall evidence for PUFA abnormalities across mental disorders is weak and heterogenous.
Other studies					
Yao et al. (183)	CS CC biomarker study (with a > 5-week medication withdrawal)	24 SCZ Med; 19 SCZ NM	Adults (M only): 20–55	↓LA and ↓AA in M and NM even after withdrawal. ↓Membrane UI	ERY ↓LA and ↓AA in both M and NM, suggesting non-medication driven PUFA abnormalities and altered membrane PL in SCZ.
Ranjekar et al. (187)	CS CC biomarker study	31 SCZ; 10 BD; 31 NC	Adults, mean age SCZ 37.32 ± 7.18, BD 40.8 ± 8.29	↓EPA and ↓DHA in SCZ and BD. ↓ALA in BD. ↓ antioxidant enzyme activity, ↑ oxidative stress markers No reduction in AA or other n-6 in SCZ.	ERY ↓n-3 PUFAs with concurrent oxidative stress and lipid peroxidation; no direct association demonstrated.
Reddy et al. (184)	CS CC biomarker study	19 FES NM; 31 NC	Adults, mean age SCZ 27.5 ± 6.3, NC 28.4 ± 7.0	↓ total PUFAs, including ↓AA (18%), ↓DPA (36%) and ↓DHA (26%). No change in saturated and monosaturated FAs.	ERY ↓PUFAs in FES NM prior to treatment, suggesting an early membrane lipid abnormality and non-medication induced effects.
Kale et al. (175)	CS CC biomarker study (ERY, PLA and CSF)	31 FEP NM (14 M + 17 F); 48 NC (27 M + 21 F)	Adults, mean age ∼31.9–35.1	ERY ↓DHA (primarily M) and ↓DPA (primarily F). No change in AA. DHA correlated with symptom severity. CSF ↑ALA, ↑EPA, ↑DHA and ↑DPA (DHA M-specific, DPA box sexes). CSF↓ AA and ↓LA (primarily M) No significant differences in PLA DHA/AA.	Opposite CSF (↑) and ERY (↓) DHA/DPA changes with unaltered PLA levels, indicating compartment-specific PUFA dysregulation in early psychosis with sex-dependent effects.
Le et al. (182)	CS CC biomarker study	72 UHR (34 F + 38 M); 18 FES (11 F + 7 M); 39 NC (21F + 18 M)	Young adults, mean age 18.9 ± 4.5 to 24.5 ± 8.1	ERY ↓EPA, ↓DPA in UHR and FES. ↑total n-6 PUFA and ↑AA in FES	ERY↓EPA/DPA may reflect early psychosis vulnerability.
McNamara et al. (178)	Prospective LS biomarker study (8 and 52 weeks)	40 FE BD (20 F + 20 M); 40 NC (20 F + 20 M)	Young adults, mean age 17.8 ± 3.8 to 18.5 ± 4.2	ERY ↓DHA at baseline and after 8- or 52-week treatment with lithium/quetiapine No changes in DPA/EPA and AA.	Early ERY ↓DHA persists despite symptom improvement, suggesting a stable abnormality not fully explained by altered DHA biosynthesis or membrane turnover.
Rice et al. (179)	Cross-sectional CC biomarker study	80 (75 M) UHR (NM); 142 NC	15–25	ERY ↓EPA, ↓ALA and ↓total n-6 PUFAs	↓n-3 PUFAs support a putative omega-3 deficiency pattern in emerging psychosis with disrupted FA metabolism.
McNamara et al. (190)	CS CC biomarker study	36 UHR BD; 30 HR; 35 FE BP; 28 NC	Adolescents, mean age 15.1 ± 2.2 to 15.9 ± 2.5	ERY ↓DHA, ↓EPA, ↓ n-3 index and↑ AA:DHA ratio in BD and UHR-BD. ↓EPA + DHA associated with greater mood severity.	↓EPA +DHA (n-3 index) before or during early BP and may represent candidate biomarkers of BP risk.
Solberg et al. (275)	LS (5-year follow-up)	Acute T1 SCZ 55; chronic SCZ T2 55; T1 20 NC; T2 51 NC; 55%–69.1% M across groups	Young to middle-aged adults, mean age 26.5 ± 6.1 to 33.0 ± 6.1	ERY ↓total PUFA and LC-PUFA at baseline T1, no difference at T2 (5 years). Serum TAGS↑ at T1 and T2. ↑serum TAGs were associated with severity and poorer functioning at T2. PUFAs were associated with symptoms at T2 but not at T1.	↑ serum TAGs may represent a stable trait marker for SCZ. Membrane PUFA abnormalities are more dynamic and variably associated with symptoms.
Berger et al. (227)	LS (7-year follow-up; Vienna omega-3 cohort)	69 UHR for psychosis (47 F, 22 M)	Adolescents, mean age 16.38 ± 1.83	ERY baseline ↑n-6:n-3 PUFA ratio and ↓total n-3 LC-PUFAs, ↓EPA and ↓DHA, predicted mood disorders over 7 years. DPA levels showed sex differences: ↓ in F.	↑n-6:n-3 PUFA ratio and ↓EPA/DHA at baseline may specifically predict later mood disorder in UHR individuals supporting potential value for risk stratification.
Berger et al. (172)	Secondary LS biomarker analysis (NEURAPRO cohort)	285 UHR for psychosis	Young adults, mean age 18.97 ± 4.49	ERY ↓DHA and ↓EPA were associated with worse clinical outcomes. ↓n-3 PUFAs (n-3 index) and ↑n-6:n-3 ratio were associated with symptom scores.	↓n-3 PUFAs and ↑n-6:n-3 ratios were related to symptom severity, supporting their potential relevance to psychosis risk prediction.
Mongan et al. (192)	LS (ALSPAC cohort)	Psychotic experiences 3,653 (61.8% F), negative symptoms score 3,484 (62.5% F)	Children and young adults: 7, 15, 17, and 24.	PLA↑ n-6:n-3 ratio and ↓DHA associated with more psychotic experiences and greater negative symptoms.	Developmental PUFA trajectories may influence psychosis-spectrum burden.

Med, on medication; NM, no medication; NC, normal control; BD, bipolar disorder; SCZ, schizophrenia; F, female; M, male; FE, first episode, FES, first-episode schizophrenia; FEP, first-episode psychosis; UHR, ultra-high risk; HR, high risk; CC, case/control; CS, cross-sectional; LS, longitudinal, UI, unsaturation index; ERY, ERY; PLA, PLA; CSF, cerebrospinal fluid; AA, arachidonic acid 20:4 n-6); DGLA, dihomogammalinolenic acid (20:3 n-6); DHA, docosahexaenoic acid (22:6 n-3); DPA, docosapentaenoic acid (22:5 n-3); EPA, eicosapentaenoic acid (20:5 n-3); LA, linoleic acid (18:2 n-6).

### 8.1 Peripheral blood biomarkers

Several studies have reported an overall reduction in erythrocyte membrane n-3 fatty acids in individuals with established schizophrenia, as well as those at ultra-high risk (UHR) of psychosis or experiencing first-episode psychosis (FEP) (171–174). More specifically, significantly reduced erythrocyte and plasma concentrations of DHA and EPA have been observed in both medicated and medication-naïve patients with schizophrenia, UHR and FEP cohorts, and individuals with bipolar disorder (171, 172, 175–182).

With respect to n-6 PUFAs, an overall reduction in erythrocyte membrane n-6 fatty acids has been reported in UHR individuals (179). More specifically, decreased concentrations of both AA and less consistently LA, have been observed in erythrocyte membranes and plasma from UHR cohorts and patients with schizophrenia, although findings vary according to medication status (171, 176, 180, 183–186). This is consistent with correlational evidence showing an inverse relationship between AA and n-6 PUFA consumption and schizophrenia incidence rates across 24 countries (56). However, other studies have reported normal peripheral concentrations of AA and LA in both schizophrenia and bipolar disorder cohorts (181, 187). Therefore, while reductions in n-3 PUFAs, particularly DHA, have been relatively consistent across psychosis-spectrum studies, the overall evidence for alterations in n-6 PUFAs, such as LA and AA, remains heterogenous.

Recent umbrella-review evidence encompassing thousands of participants similarly concluded that reductions in circulating n-3 LC-PUFAs represent the most robust lipid abnormality in schizophrenia and bipolar disorder, whereas evidence for n-6 PUFA alterations remains inconclusive (188). However, the overall evidence base was considered weak, underscoring the need for further research for pediatric and adolescent populations, particularly those with mood disorders (188). Consistent with these findings, a large meta-analysis of FEP reported significantly lower erythrocyte DHA concentrations relative to healthy controls, further supporting reduced DHA as one of the most reproducible lipid abnormalities across the psychosis spectrum, and one of the largest effect sizes among the oxidative and inflammatory markers examined (189).

Evidence increasingly points to the importance of balance between n-6 and n-3, and overall, this may be more informative than absolute n-3 and n-6 PUFA concentrations alone. Of the few studies that have measured this, Sethom et al. (185) observed an increased erythrocyte membrane n-6:n-3 ratio in patients with schizophrenia, despite lower overall levels of n-3 and n-6 PUFAs. Higher erythrocyte membrane AA:EPA ratios were also observed in UHR for psychosis and bipolar disorder patients in manic phase (171, 190), and a higher AA:DHA ratio was found in bipolar I disorder (190), indicating that imbalance of n-6 to n-3 PUFAs specifically is associated with psychotic disease status.

Few population-based longitudinal studies have investigated PUFA status across development, limiting our understanding of how changes in PUFA levels influence psychosis risk over time. Among the available studies, findings are mixed. While Thompson et al. (191) found little evidence that single childhood or adolescent PUFA measurements predict later psychosis outcomes in the Avon Longitudinal Study of Parents and Children (ALSPAC) cohort, Mongan et al. (192) showed that persistently lower DHA levels and higher n-6:n-3 ratios across development were associated with increased psychosis risk, highlighting the importance of longitudinal trajectories.

A limited number of studies, most involving relatively small clinical cohorts, have examined associations between PUFA status and specific dimensions of psychotic and affective symptomatology. An inverse relationship has been reported between PUFA levels, particularly DHA, and negative symptoms including anhedonia, avolition, alogia, and blunted affect in unmedicated individuals with schizophrenia (185). In FEP, higher baseline DHA and EPA levels were significantly associated with better social cognition performance, whereas higher AA levels were associated with increased hostility (193). In bipolar disorder, higher plasma concentrations of the n-6 LC-PUFA DGLA were associated with greater neuroticism, depressive symptoms, and poorer functional outcomes, whereas higher concentrations of the SC-PUFAs such as LA and ALA were associated with lower neuroticism and improved functioning, suggesting potentially protective effects of SC-PUFAs (173).

### 8.2 Cerebrospinal fluid and post-mortem brain studies

As discussed above, peripheral studies of plasma and erythrocyte membrane PUFA status have demonstrated altered DHA, EPA, AA levels and n-6:n-3 ratios across the psychosis spectrum, some of which appear to partially normalize following antipsychotic treatment (181, 182). Extending these observations to the brain, McNamara et al. (194) reported a 20% reduction in DHA, accompanied by an elevated AA:DHA ratio, in the post-mortem orbitofrontal cortex of individuals with schizophrenia. These changes were most pronounced in males, suggesting sex-specific alterations in cortical PUFA metabolism. Similarly, orbitofrontal cortex DHA and AA were reduced by 24% and 14%, respectively, in bipolar disorder, independent of sex or age. Taha et al. (195) also reported a 20% reduction in DHA within total prefrontal cortex glycerophospholipids in schizophrenia, accompanied by lower fractional concentrations of both DHA and AA, indicating altered cortical PUFA composition.

However, post-mortem findings have been less consistent than those from peripheral studies. This variability likely reflects differences in brain regions examined, lipid fractions and species analyzed, methodological approaches, and the generally small sample sizes of post-mortem studies. For example, Igarashi et al. (196) found no evidence of reduced DHA or AA in the prefrontal cortex of individuals with bipolar disorder and observed no differences in total glycerophospholipids, the principal membrane phospholipids in which PUFAs are esterified, or in individual phospholipid classes, including phosphatidylcholines (PCs), phosphatidylethanolamines (PEs), phosphatidylinositols (PIs), phosphatidylserines (PSs), and plasmalogens (PC-P and PE-P). Likewise, Hamazaki et al. (197) found no significant alterations in n-3 PUFA species, although both schizophrenia and bipolar disorder were associated with reduced n-6 DPA-containing PC and PS species, while AA-containing PC species were selectively reduced in schizophrenia. These findings shifted attention from individual PUFA concentrations toward alterations in phospholipid composition and membrane lipid remodeling. Consistent with this, Hamazaki et al. (198) subsequently reported no significant differences in individual LC-PUFAs, including DHA, in the corpus callosum of patients with schizophrenia or bipolar disorder. Similarly, another study demonstrated altered membrane lipid composition in prefrontal white matter, including reduced PC concentrations in bipolar disorder and lower DPA content within PC and PE fractions in both disorders (199). Beasley et al. (200) later found no robust differences in phospholipid or fatty acid composition in the dorsolateral prefrontal cortex of patients with schizophrenia or bipolar disorder after correction for multiple testing. However, several n-6 PUFAs, including DGLA, AA, and DPA, were positively associated with reelin expression, suggesting a link between lipid metabolism and neurodevelopmental and synaptic signaling pathways.

The extent to which peripheral fatty acid measures reflect central nervous system PUFA composition remains uncertain. Notably, Kale et al. (175) reported increased CSF concentrations of several n-3 PUFAs, including DPA, ALA, and EPA, as well as elevated DHA levels in males with first-episode psychosis. This contrasted with reduced erythrocyte membrane and plasma PUFA concentrations observed in the same cohort, highlighting a potential dissociation between peripheral and central PUFA profiles. Although these findings caution against assuming a direct correspondence between peripheral and central measures, peripheral lipid profiles may nonetheless retain value as accessible biomarkers of systemic PUFA metabolism and dietary intake, while also reflecting biologically meaningful states relevant to disease risk and progression. Overall, evidence from post-mortem brain tissue and CSF suggests that disturbances in phospholipid remodeling and PUFA metabolism may represent more consistent features of psychotic disorders than reductions in brain DHA alone.

### 8.3 Lipidomics studies

While targeted analyses of PUFAs provide important insight into lipid abnormalities in psychosis, they capture only a small fraction of the lipidome. Bioactive lipids are increasingly recognized as key structural and signaling molecules involved in membrane function, inflammation and neuronal communication, making them highly relevant to neuropsychiatric disorders (74, 201–203). Consequently, lipidomics offers the potential to move beyond isolated PUFA measurements and characterize broader lipid signaling and remodeling pathways.

Although lipidomics has been increasingly applied to schizophrenia and bipolar disorder, most studies have focused on biomarker discovery rather than disease mechanisms (204, 205). Nevertheless, findings consistently demonstrate lipid perturbations extending beyond isolated PUFA abnormalities. For example, a large-scale multicohort untargeted plasma lipidomics study identified shared alterations across schizophrenia, bipolar disorder and depression, supporting transdiagnostic lipid pathways (206). Notably, schizophrenia showed the most extensive lipid dysregulation characterized by increased PCs, triglycerides (TAGs), ceramides (Cers), alongside reduced acylcarnitines (CARs) and plasmalogens (PC-Ps), highlighting widespread disturbances in lipid metabolism.

Additional studies have identified distinct disorder- and sex-specific lipidomics signatures involving glycerophospholipids, sphingolipids, plasmalogens and PUFA-containing lipids, highlighting their potential utility for diagnosis and patient stratification (207–209). Recent reviews further report recurring lipidomics patterns across studies, including depleted plasmalogens and acylcarnitines and elevated TAGs in both schizophrenia and bipolar disorder, whereas ceramide alterations may be more specific to bipolar disorder (210). Similarly, Modesti et al. (203) highlighted overlapping abnormalities in phospholipids, fatty acids and triacylglycerols across schizophrenia, bipolar disorder and depression, alongside convergence with lipid-related genomics signals, supporting shared metabolic pathways that transcend traditional diagnostic boundaries.

Beyond biomarker discovery, a smaller number of studies have explored the biological significance of lipid alterations. Post-mortem findings have linked altered phospholipid composition to dysregulated membrane remodeling and identified associations between n-6 PUFAs and reelin signaling, implicating lipid dysregulation in neurodevelopmental and synaptic signaling pathways (199, 200). More recently, integrative omics approaches have linked lipid alterations to other molecular features of psychosis. Sano et al. (211) associated a specific AA-containing PI species (PI 16:0/20:4) with the *TNC* gene, APOA1 and PON2 proteins, suggesting interactions between phospholipid metabolism, cholesterol regulation and oxidative stress in schizophrenia; while another study linked early plasma glycerophospholipids (PC and lyso PC) and protein alterations to later psychosis risk in the ALSPAC cohort (212). However, these findings remain largely associative and hypothesis-generating.

### 8.4 Methodological considerations and limitations

Several important limitations need to be considered when linking PUFA status to psychotic disorders. Many studies are relatively small, which limits their statistical power and generalisability. In addition, methodological heterogeneity is common, including differences in recruitment strategies, with some cross-sectional studies exhibiting substantial differences in case and control demographics. A further limitation is the frequent absence of detailed dietary intake assessment, which may influence PUFA profiles and limits the ability to link observed peripheral PUFA levels to intake. There are also important biological and methodological considerations relating to the interpretation of PUFA measures across different compartments. Marked differences between erythrocyte membrane, plasma, and CSF PUFA concentrations have been reported, as illustrated by studies such as Kale et al. where CSF findings contrasted with peripheral blood measures (175). While plasma PUFA levels are considered more sensitive to short-term dietary intake, erythrocyte membrane measures are generally viewed as more stable indicators of longer-term status, reflecting approximately 3 months of intake (213). Nonetheless, while plasma and erythrocyte measures are thought to be reasonably correlated (214), both are influenced by a range of environmental and behavioral factors and can be considered relatively dynamic measures.

Across the literature, many potential confounders remain insufficiently accounted for, including dietary composition, cannabis use, cigarette smoking and medication status. These factors may independently influence both nutrient intake and metabolism as well as and psychiatric risk, complicating causal inferences. Additional limitations are particularly evident in longitudinal cohorts, where relatively low numbers of incident psychosis cases reduce statistical power, and attrition or missing data further impact robustness. Inconsistencies in methodological protocols, including whether blood samples were collected in fasting or non-fasting states, may introduce additional variability across studies.

With regard to lipidomics, methodological heterogeneity, incomplete control of confounders, and the predominance of untargeted, biomarker-focused studies limit mechanistic insight and comparability across the literature (204, 210). More robust, standardized and integrative lipidomics approaches, applied to relevant tissues, are needed to place PUFA abnormalities within the broader lipidome and elucidate disease-relevant pathways in schizophrenia and bipolar disorder. Jointly, these limitations highlight the need for larger, methodologically standardized longitudinal studies with integrated dietary, biological, and environmental assessments to clarify the role of PUFAs in the development and progression of psychotic disorders.

## 9 Genomic evidence linking polyunsaturated fatty acid metabolism and psychotic disorders

While observational studies are susceptible to confounding and reverse causation, genomic approaches provide a powerful means of interrogating causal relationships. By exploiting genetic variants that influence PUFA biosynthesis, genome-wide association studies (GWAS) and Mendelian randomisation (MR) studies have begun to clarify whether altered PUFA metabolism contributes directly to psychiatric risk. MR is particularly valuable in nutritional psychiatry, where research is often limited by biological complexity, confounding, and small effect sizes of individual nutrients. This approach offers considerable potential for identifying causal relationships between nutritional exposures and psychiatric outcomes, thereby strengthening the evidence base from which effective interventions may be developed (215).

### 9.1 Bipolar disorder studies

Genome-wide association studies have implicated variants within the *FADS* gene cluster in bipolar disorder risk, firstly within a Japanese ancestry cohort (57) and subsequently in two large scale GWAS of individuals of European ancestry (5, 54), one of which pinpointed *FADS2* specifically. Integrative analyses further support a functional role for common *FADS* cluster variants. MR studies have leveraged naturally occurring genetic variation in PUFA metabolism to examine whether lifelong differences in PUFA synthesis and availability influence risk of psychiatric disorders. Stahl et al. (54) identified associations between bipolar disorder and DNA methylation at CpG sites within *FADS2* in both brain and blood, implicating epigenetic regulation of this pathway. Furthermore, Ikeda et al. (57) reported that risk-associated variants across the *FADS1/2/3* cluster act as expression quantitative trait loci (eQTLs), influencing *FADS1* expression in the cerebellum and cerebral cortex, and *FADS2* expression in the temporal cortex. Therefore, common variation in the *FADS* locus may contribute to bipolar disorder risk by modulating lipid metabolism through coordinated effects on gene expression and epigenetic regulation.

Further evidence comes from a two-sample metabolome-wide MR study that integrated data on over 900 plasma metabolites (from approximately 14,000 individuals of European ancestry) with the latest genome-wide association summary statistics from around 42,000 cases of bipolar disorder. The study identified significant associations between bipolar disorder and AA levels, as well as several lipid species containing AA or LA fatty acid side chains (216). Lower circulating levels of these lipids were associated with increased disease risk. Notably, these associations were driven by genetic variants within the FADS gene cluster. In agreement, two other MR studies revealed a protective effect of n-3 in bipolar disorder, whereby higher n-3% was associated with a reduced risk of bipolar disorder and conversely higher n-6 increased risk (217, 218). Xu et al. demonstrated that these effects were again mediated by FADS1/2 genomic variants (217). These findings strengthen the emerging view that altered PUFA metabolism, particularly FADS-dependent conversion of LA to AA, may represent a biologically important pathway in bipolar disorder, although the specific mechanisms linking this pathway to disease risk remain to be elucidated.

### 9.2 Schizophrenia studies

Despite substantial genetic overlap between schizophrenia and bipolar disorder (8), significant genome-wide associations between *FADS* variants and schizophrenia have not been reported. However, converging evidence from MR studies implicates PUFA-related genetic variants, many of which map to the *FADS* region, in schizophrenia risk. In a two-sample MR study, Jones et al. (55) analyzed schizophrenia GWAS data from European and Asian ancestry populations and found that higher levels of the SC-PUFAs LA (n-6) and ALA (n-3) were associated with increased schizophrenia risk, whereas higher levels of LC-PUFAs including EPA, DHA, DPA, and AA were protective, with the strongest effects observed for DHA. For most fatty acids, these associations were driven by variants within the *FADS* gene cluster, while contributions from *ELOVL2* were weaker. The relative contributions of n-3 and n-6 pathways could not be disentangled due to lack of measures on n-6:n-3 ratios.

A further MR similarly reported protective effects of genetically predicted higher levels of n-6 LC-PUFAs AA and gamma-linoleic acid (GLA) on schizophrenia risk, whereas short-chain precursors LA and ALA were associated with increased risk (56). No significant effects were observed for n-3 PUFAs including DHA. These associations were primarily driven by variants within the *FADS* locus, particularly in *FADS1*, with additional contribution from in *NTAN1/PDXDC1*, a locus implicated in fatty acid metabolism (56). However, it should be noted that these analyses were restricted to European ancestry populations. Moreover, another two-sample metabolome-wide MR study that observed associations between AA-containing lipids and bipolar disorder failed to reproduce such associations for schizophrenia. While some metabolites were linked to schizophrenia risk, effect sizes were substantially smaller, suggesting a more prominent role for lipid dysregulation in bipolar disorder (216).

Across bipolar disorder and schizophrenia, converging evidence therefore implicates impaired conversion of SC to LC-PUFAs as a shared disease mechanism, mediated by variation in the *FADS* gene cluster. Importantly, these findings are broadly consistent with observational studies reporting that lower peripheral LC-PUFA levels are associated with disease risk and suggest a common underlying mechanism in PUFA biosynthesis via impaired elongation and desaturation of dietary precursors, with disorder-specific differences in downstream effects and lipid signaling pathways. It remains unclear whether risk is driven primarily by an excess of SC-PUFAs [which can exert biological effects in and of themselves; (86, 91)] due to a bottleneck in conversion, deficiency of LC- PUFAs, or both. Additionally, important questions remain regarding the relative contributions of n-3 and n-6 pathways, the influence of dietary exposures and differing genetic backgrounds, and the precise molecular mechanisms through which disrupted lipid metabolism affects disease mechanisms.

While these studies point toward impaired PUFA biosynthesis as a potential upstream mechanism, the downstream consequences remain poorly understood. Notably, one MR study tested whether inflammatory pathways mediated the association between PUFAs and schizophrenia risk but found no evidence of mediation via C-reactive protein (CRP), suggesting that PUFA-related effects on inflammatory markers may not account for these associations (56). Given the broad biological roles of PUFAs, alterations in their synthesis may influence a wide range of neurobiological pathways implicated in psychosis. Further research is needed to clarify the cellular, molecular, and functional consequences of FADS-mediated variation in PUFA metabolism, particularly in relation to neurodevelopmental processes, neurotransmission, and immune signaling. Consistent with an upstream role for PUFA metabolism, reverse MR analyses have found little evidence that genetic liability to psychiatric disorders alters circulating PUFA levels (217), supporting the interpretation that altered PUFA metabolism is more likely to contribute to disease susceptibility than arise as a consequence of disease.

### 9.3 Limitations and methodological considerations

Several limitations of the genomic evidence outlined should be considered. Although findings are beginning to emerge across multiple ancestries, much of the current evidence derives from populations of European ancestry. Given well-established population differences in *FADS* allele frequencies, including variants such as rs174537 that strongly influence desaturase activity and circulating PUFA levels, ancestry-specific variation in LC-PUFA biosynthetic capacity is likely to be important (219). In addition, GWAS and MR studies capture the effects of lifelong genetic predisposition and may therefore be less sensitive to influences operating discretely during critical developmental windows, such as gestation or adolescence. Moreover, in some MR studies, PUFA genetic instruments (genetic variants used as proxies for lifelong PUFA exposure) were derived from plasma rather than membrane lipid measures, which asides from being highly dynamic, may not accurately reflect the biologically relevant PUFA pools involved in membrane function and signaling. Furthermore, although the studies discussed assessed for weak instrument bias, the limited strength of some genetic instruments restricted analyses of individual PUFA species and n-6:n-3 ratios. Larger genome-wide association studies identifying genetic determinants of n-3 and n-6 LC-PUFA concentrations, particularly EPA and AA, are needed to improve the precision of MR estimates and disentangle the potentially distinct or opposing effects of individual fatty acids, which remain difficult to resolve because of their shared metabolic pathways and correlated genetic architecture.

Nonetheless, findings from GWAS and MR analyses provide converging evidence that genetically determined variation in PUFA metabolism contributes to disease risk and broadly corroborate observational studies linking altered PUFA metabolism to schizophrenia and bipolar disorder. Although effect sizes differ between disorders, many findings implicate pathways governing the conversion of SC precursors into LC-PUFAs, particularly variation within the *FADS* gene cluster. These studies suggest that modifying PUFA status through nutritional or pharmacological intervention may represent a viable strategy for prevention or treatment, a hypothesis that has been addressed in a series of clinical supplementation studies.

## 10 Clinical evidence for omega-3 polyunsaturated fatty acid supplementation in psychotic disorders

Given the accumulating evidence implicating PUFA metabolism in psychotic disorders, together with the neuroprotective and immunomodulatory properties of n-3 PUFAs and their favorable safety profile as a relatively low-cost intervention (220), considerable interest has emerged in targeting PUFA pathways as a therapeutic strategy. This has prompted a series of studies, including observational, longitudinal and randomized clinical trials (RCTs) investigating whether n-3 fatty acid supplementation can improve outcomes in psychotic disorders when used alongside standard treatments. Here, we summarize the evidence from these studies, which are additionally summarized in Table 2.

**TABLE 2:** Summary of key human intervention studies of n-3 polyunsaturated fatty acid (PUFA) in psychotic disorders included in this review.

References	Design and duration	Population	Age (years)	Intervention	Findings	Conclusions
Meta-analyses						
Chen et al. (237)	Meta-analysis of 10 RCT studies	SCZ 559 (prodromal, FEP, relapse, acute exacerbation and chronic)	Varied across studies	Varied ∼1–3 g/d across studies (EPA ± DHA)	Improved outcomes in prodromal/FEP; limited/mixed effects in chronic SCZ; not recommended during acute exacerbation/antipsychotic discontinuation.	Stage-specific benefits, greatest in early psychosis, with limited or mixed benefit in chronic SCZ.
Chen et al. (238)	Network meta-analyses of 21 studies (9 RCTs, 12 observational) at 6, 12, ≥24 months	SCZ 1983 (M mean 54.5% ± 14.9%) at UHR/CHR for psychosis	Mean 20.2 ± 2.59	Varied across included RCTs (EPA ± DHA)	Lowest transition risk with n-3 PUFAs at 6, 12, and ≥24 months; no added benefit with psychotherapy.	Supports n-3 PUFAs for psychosis intervention in CHR individuals; further RCTs required.
Lin et al. (235)	Systematic review and meta-analysis of 16 RCTs	SCZ FEP/UHR, 1,435	Varied	Varied ∼0.3–3 g/d (EPA ± DHA)	No overall benefit in SCZ/UHR; possible benefit in FEP, ≥24-week treatment or adjunctive antioxidants.	No overall support for routine n-3 supplementation; possible benefit in early-stage SCZ and with longer treatment.
Other studies						
Stoll et al. (239)	DB, PC RCT; 4 months	BD 30 (10 M + 20 F): n-3 14; placebo 16	18–65 (mean 44)	EPA 6.2 g and DHA 3.4 g/d fish oil adjunctive to usual treatment vs. olive oil placebo	↑ remission/survival time; improved clinical outcomes (depression, but not mania) vs. placebo; well-tolerated.	Preliminary evidence supporting adjunctive n-3s in BP.
Peet et al. (221)	DB, PC RCT; 3 months	SCZ 45 (30 M + 15 F): EPA 15; 16 DHA; placebo 14	Mean age 42.0 ± 10.6 to 44.2 ± 11.3	Adjunctive 2 g/d EPA, 2 g/d DHA vs. corn oil placebo	↓ psychotic symptom severity; EPA beneficial DHA ineffective; well-tolerated.	Preliminary evidence that EPA may improve SCZ symptoms as an adjunctive treatment.
Frangou et al. (240)	DB, PC RCT; 12 weeks	BD 75 (18 M + 57 F): 1 g/d EPA 24; 2 g/d EPA 25; placebo 26	Mean 45.5 ± 9.6 to 49.2 ± 11.7	Adjunctive ethyl-EPA 1 or 2 g/d vs. paraffin placebo	↓ depressive symptoms with adjunctive ethyl-EPA; no effect on mania; well-tolerated.	Ethyl-EPA may benefit BP, particularly depressive symptoms.
Amminger et al. (222)	DB, PC RCT; 12 weeks + 40-week follow-up (Vienna High Risk study)	SCZ UHR 81 (51 M + 30 F): n-3 41; placebo 40	Mean 16.0 ± 1.7 to 16.8 ± 2.4	n-3 PUFAs 1.2 g/day (containing 700 mg EPA, 480 mg DHA) vs. placebo	↓ psychosis transition; improved symptoms and functioning; sustained post-treatment benefits; ↓ERY n-6:n-3 ratio linked to functional improvement.	Supports potential long-term preventive/ neuroprotective effects of n-3 PUFAs.
McGorry et al. (223)	DB, PC multicentre RCT; 6 months + 6-month follow-up (NEURAPRO)	SCZ UHR 304 (139 M + 165 F): n-3 153; placebo 151	13–40 (mean 19.1 ± 4.6)	n-3 PUFAs 1.4 g/day + cognitive-behavioral case management and antidepressants vs. placebo (paraffin oil)	No significant difference in transition rates, symptoms or functioning between groups.	No added benefit of n-3s over intensive psychosocial treatment.
McPhilemy et al. (242)	DB, PC RCT; 52 weeks	80 BD euthymic/remitted; prophylaxis study (39 M + 41 F): n-3 (1 g/d EPA + 1 g/d DHA) 40; Placebo 40	Mean 45 ± 12 to 48 ± 13	Adjunctive n-3 (1 g/d EPA + 1 g/d DHA) vs. placebo	No significant effects on relapse or overall symptoms; slight reduction in hypomania scores.	No prophylactic benefit for depressive or manic relapse in BD.
Saunders et al. (244)	DB, PC RCT; 48 weeks	82 BD (10 M + 72 F): n-3 41; placebo 41	Mean 42.7 ± 13.7 to 44.3 ± 14.2	High n-3 + low n-6 diet vs. control diet (12 weeks)	Improved mood symptoms;↑ ERY n-3 status, ERY↓ n-6:n-3 ratio	High n-3/low n-6 dietary intervention may improve mood outcomes in BD.
Qurashi et al. (224)	DB, PC multicentre RCT; 12 months [6 months + 6-month follow-up; (NAYAB study)]	326 SCZ ARMS (134 F + 192 M) n-3 82; minocycline; n-3 + minocycline 82; placebo 82	16–35	1.2 g/d n-3 PUFAs (EPA 720 mg + DHA 480 mg) for 6 months; minocycline: 100 mg/day (week 1), then 200 mg/day thereafter vs. placebo	No reduction in psychosis transition; improved ARMS/depressive symptoms; n-3 associated with a near-significant trend for ↑transition (underpowered study).	No evidence of reduced psychosis transition but improved ARMS symptom severity and mood.
Winter-Van Rossum et al. (225)	DB, PC multicentre RCT; 6-month intervention + 18-month follow-up (PURPOSE study)	SCZ 135 UHR (75 F + 30 M); n-3 67; placebo 68	13–20 (mean 15.5 ± 1.8 to 15.8 ± 1.7)	Adjunctive 1.2 mg/d (720 mg EPA + 480 mg DHA) vs. placebo + treatment as usual	No benefit for psychosis transition, symptoms, functioning, or cognition (2 years follow-up).	Adjunctive n-3 PUFAs conferred no additional benefit over standard care in UHR individuals.

ARMS, at-risk mental state; CC, case/control; CHR, clinical high risk; DB, double-blinded; FE, first episode; FES, first-episode schizophrenia; FEP, first-episode psychosis; F, female; M, male; HR, high risk; SCZ, schizophrenia; UHR, ultra-high risk.

### 10.1 Evidence for n-3 PUFA supplementation in schizophrenia: clinical and observational studies

Overall, findings from RCTs have been mixed. Several early RCTs, including those by Peet et al. (221), Amminger et al. (221, 222) reported beneficial effects of n-3 PUFA supplementation in the Vienna High Risk trial. However, subsequent large-scale multicentre trials, including NEURAPRO [McGorry et al. (223], NAYAB [Qurashi et al. (224)], and PURPOSE [Winter-Van Rossum et al. (225)], have generally failed to demonstrate significant clinical benefit. However, it is important to note that a substantial heterogeneity in study design represents an important limitation of the current literature and may account, at least in part, for the inconsistent results observed across trials. The early Vienna High Risk (VHR) Study was a single-center RCT that included 81 UHR participants, used a 12-week treatment window, and reported outcomes over a 6.7-year follow-up period. In contrast, subsequent studies including NEURAPRO, NAYAB and PURPOSE were larger multicentre trials comprising 304, 326, and 135 participants, respectively (223–225). Participants in these trials were all classed as UHR, except for the NAYAB trial, which instead classified participants as “at risk mental state.” These trials typically employed longer treatment durations of at least 6 months, but shorter follow-up periods.

Secondary analyses of both the VHR and NEURAPRO studies have identified consistent findings. Amminger et al. (226) reported that increases in the erythrocyte n-3 index between baseline and follow-up predicted less severe psychopathology and improved functional outcomes within the NEURAPRO cohort, independent of treatment allocation (226). Similarly, follow-up analyses of the VHR cohort demonstrated that erythrocyte membrane n-3 levels were associated with a lower risk of mood disorders during follow-up, regardless of treatment arm, and higher n-6:n-3 ratios at baseline in UHR participants predicted development of mood disorders over the 7-year follow up (227). Secondary analyses of baseline and 6-month follow-up biomarkers from the NEURAPRO trial have provided further insight into potential mechanisms underlying the effects of omega-3 supplementation. Analysis of inflammatory cytokines by Susai et al. (110) found that increases in n-3 fatty acid levels were associated with a predominantly anti-inflammatory plasma profile in UHR individuals. However, these changes were not associated with improved clinical outcomes at either 6- or 12-month follow-up. In contrast, subsequent proteomic analyses identified complement and coagulation proteins as potential mediators of the relationship between changes in n-3 PUFA levels and functional outcomes. Specifically, increases in n-3 PUFAs were associated with reduced symptom severity and improved cognitive performance through effects on complement and coagulation pathways (228). Together, these findings suggest that the clinical effects of n-3 PUFAs in the UHR state may be mediated less by broad suppression of inflammatory cytokines and more by modulation of specific immune signaling pathways. However, it should be noted that participants classified as UHR in the VHR study developed a range of psychiatric outcomes, including mood disorders, and analyses performed independently of treatment allocation effectively pool the randomized groups. Consequently, these secondary analyses are observational in nature and cannot establish causal treatment effects.

Other interventions have produced similarly mixed results. The NAPLS prodromal longitudinal study included an RCT component that concluded n-3 supplementation had no significant effect compared with placebo (229). However, as with NEURAPRO, PURPOSE, and NAYAB, relatively low transition rates may have limited the study statistical power. Because statistical power in UHR cohorts is strongly dependent on the number of transition events observed, lower-than-expected transition rates reduce the ability to detect clinically meaningful differences between treatment groups, even when true effects may be present. Subsequent analyses of the NAPLS cohort reported that lower erythrocyte PUFA levels were associated with poorer functioning and increasing symptom severity prior to psychosis onset (230).

A methodological exception is the PORT study (231), a prospective observational study in which food frequency questionnaire data was collected from 62 UHR individuals and 33 healthy controls over a 12-month period, in the absence of supplementation intervention. The study concluded that higher n-6 intake, and consequently a higher dietary n-6 ratio, was associated with increased transition to psychosis. These findings provide additional support for the hypothesis that both absolute PUFA intake and the balance between n-6 and n-3 fatty acids may influence psychosis risk.

Meta-analytic evidence of omega-3 PUFA supplementation in psychotic disorders is also consistent with the heterogeneity observed across individual trials. Pooled analyses generally indicate small and statistically non-significant effects of n-3 supplementation on primary psychosis outcomes, with some analyses suggesting modest benefits particularly in shorter-duration and EPA-enriched interventions (232–236). However, these effects are not robust across sensitivity analyses and are substantially attenuated when larger, more recent multicentre trials are included. Meta-analyses by Chen et al. (237) indicated disease-stage specific effects however, with n-3 supplementation reducing positive symptom severity and decreasing conversion rates in FEP participants, meanwhile patients with established disease showed little benefit. This observation is supported by more recent meta-analyses in which n-3 treatment was associated with a lower rate of transition to psychosis in CHR populations (238) and benefits in FEP participants (235). Across meta-analyses, between-study heterogeneity remains high and is largely driven by variation in diagnostic stage, baseline symptom severity and PUFA levels, supplement EPA:DHA composition, dosage, and treatment duration, as well as differences in outcome measures and adjunctive treatments (236). Collectively, these findings reinforce the conclusion that although biological plausibility for n-3 PUFAs supplementation as a therapy in psychosis remains strong, current aggregated clinical evidence does not support a consistent or clinically meaningful treatment effect, at least in patients with chronic disease. n-3 PUFA supplementation may hold promise for reducing symptom severity and the risk of transition to psychosis in UHR populations; however, the current evidence remains inconclusive and further studies are needed.

### 10.2 Evidence for n-3 PUFA supplementation in bipolar disorder: clinical and observational studies

Similar trends are observed within the bipolar disorder literature. Early studies by Stoll et al. (239), Frangou et al. (240), with intervention periods of 16 and 12 weeks, respectively, reported improvements in Clinical Global Impression (CGI), Global Assessment Scale (GAS), Hamilton Depression Rating Scale (HAM-D), and Young Mania Rating Scale (YMRS) scores. In contrast, later bipolar disorder trials ranging from 12 to 52 weeks generally failed to demonstrate significant clinical benefits of n-3 supplementation (241–244). A recent RCT found n-3 supplementation to be beneficial in preventing recurrence of bipolar depression over a 6-month intervention period, though sample sizes were small (245). Two studies have reported beneficial effects upon hypomania as measured by the YMRS (242, 246). Another follow-up study by Frangou et al. (247) reported preliminary evidence that 2 g/day ethyl-EPA supplementation increased N-acetylaspartate (NAA) concentrations following a 12-week intervention in an all-female, medication-tapered cohort, suggesting a potential neurobiological mechanism of action.

In summary, RCTs and observational studies of n-3 PUFA supplementation in schizophrenia and bipolar disorder have yielded heterogeneous findings, with limited evidence for consistent clinical benefit across studies. Nevertheless, some more nuanced analyses have suggested potential improvements in symptom severity and possible reductions in transition to psychosis, particularly in individuals at UHR. While the current evidence does not provide definitive support for n-3 supplementation as an effective treatment, neither does it negate the substantial biological rationale underpinning its investigation. Rather, the inconsistency of findings likely reflects the methodological and conceptual challenges inherent to nutritional intervention studies, including variability in baseline PUFA status, genetic factors influencing PUFA metabolism, treatment timing, and intervention type.

## 11 From mechanism to intervention: challenges in translating omega-3 research

Observational, epidemiological, preclinical, and genomic evidence broadly support a role for inadequate n-3 PUFA availability in increasing vulnerability to psychotic disorders. However, as noted above, clinical trials of n-3 supplementation have yielded inconsistent and often modest effects. These mixed findings may reflect substantial heterogeneity in study design rather than a true absence of biological efficacy. It is perhaps not surprising that n-3 supplementation trials in psychotic disorders have faced many of the same methodological challenges that have complicated interpretation of the equivalent literature on neurodegenerative disorders (248). In the following section, we discuss several of these factors in turn.

Studies have employed a wide range of supplementation regimens, varying in dose, duration, chemical formulation, and the relative proportions of EPA, DHA, and other LC-PUFAs. For example, a wide range of n-3 formulations and sources are evident; several utilized fish oil preparations (239, 242, 243), whereas others administered purified ethyl-EPA (240, 241) or algal-derived DHA (249), while others focused primarily on dietary modification (244). Consequently, there is little consistency in either PUFA composition or dosage across studies, making direct comparison difficult. Such differences are likely to be important given that EPA and DHA exert overlapping but distinct biological effects, including differential influences on inflammatory pathways, membrane composition, neurotransmission, and specialized pro-resolving mediator production. Furthermore, the Stoll et al. (239) trial employed olive oil as a placebo, which may not be biologically inert given its recognized anti-inflammatory properties (250). In addition, blinding is difficult to maintain due to the distinctive properties of fish oil preparations, which can lead to partial unblinding and placebo effects.

Another caveat is that studies have often used relatively short intervention periods, for example, only 3 months in the VHR study. Short-term supplementation may be an insufficient strategy for correcting chronic or even life-long deficiencies in PUFA metabolism, as may be associated with certain *FADS* genotypes. Longer intervention periods may be advantageous when investigating n-3 PUFAs, as incorporation of LC-PUFAs into membrane phospholipids and subsequent effects on lipid signaling, inflammation, and neuronal function are likely to -up periods may occur gradually. Conversely, shorter follow reduce attrition and minimize the influence of post-trial interventions and environmental factors that could obscure treatment-specific effects.

Another major limitation is that relatively few studies have incorporated baseline or post-treatment measurements of circulating PUFA levels. This is problematic for several reasons. Firstly, this ties into a major challenge for nutritional intervention studies; controlling for the free-living nature of participants and the substantial variation in background diet. Both the NAPLS and PORT studies relied on food frequency questionnaires and self-reported dietary information. Although widely used in nutritional epidemiology, such approaches are recognized sources of measurement error and bias (67, 251). Furthermore, dietary questionnaires estimate food consumption rather than real biological exposure, providing no direct information on fatty acid absorption, tissue incorporation, or metabolism. This limitation may be particularly important in PUFA research, where circulating and membrane fatty acid levels are influenced not only by dietary intake but also by individual differences in metabolism, genetic factors, and background dietary composition. Consequently, estimated PUFA intake derived from questionnaires may not accurately reflect physiological PUFA status or downstream lipid mediator production, potentially obscuring true associations with clinical outcomes. Therefore, assessment of blood PUFA status is important not only for monitoring dietary intake but also supplement adherence.

Inclusion of baseline and follow up blood PUFA levels could also aid in stratification of subgroups with low baseline n-3 status or high n-6:n-3 ratios, who may conceivably derive the greatest benefit from supplementation. Failure to account for baseline PUFA status may contribute substantially to between-study variability, particularly in populations where dietary n-3 intake differs markedly. This is especially important because, unlike in pharmaceutical trials, PUFAs are naturally occurring compounds that will always be present within the control group. A notable exception is studies by McPhilemy et al. (242), Saunders et al. (244) which measured circulating PUFA levels, while Saunders et al. additionally quantified downstream oxylipin metabolites from baseline to 48-week follow-up. This study further stands out through comprehensive dietary assessment and regular dietitian-led counseling sessions. Together with erythrocyte fatty acid profiling and metabolite analyses, these measures provide substantially greater insight into the biological effects of dietary intake than food frequency questionnaires alone. As such, this study offers a useful framework for future trials incorporating detailed nutritional phenotyping, lipidomic profiling, and longitudinal biomarker assessment alongside clinical outcomes.

Another factor is participant demographics. Emerging evidence suggests that n-3 metabolism and tissue incorporation may differ between males and females, yet sex-stratified analyses are rarely performed. Marked differences are evident in sex distribution of trial participants, ranging from approximately 67% female participants in the VHR trial to 66% male participants in the NAYAB trial, while NEURAPRO and PURPOSE recruited more balanced cohorts (approximately 53%–55% female). Several bipolar cohorts were also heavily female-weighted (239, 240), while other investigations recruited exclusively female participants (247, 249). As females of reproductive age generally exhibit greater endogenous conversion and tissue incorporation of n-3 PUFAs than males, differences in sex composition may influence treatment response and complicate comparisons across studies (28).

A major limitation of existing RCTs is the failure to account for genetic variation in PUFA metabolism, particularly within the *FADS* gene cluster. Increasing evidence suggests that the response to n-3 supplementation is influenced by gene–diet interactions, such that individuals with different genetic capacities for LC-PUFA biosynthesis may respond differently to supplementation, particularly in the context of varying dietary n-6:n-3 ratios. Future trials incorporating genomic and lipidomic measures will therefore be better positioned to identify biologically relevant subgroups most likely to benefit from n-3 interventions.

A broader challenge is the inherently pleiotropic nature of PUFA biology. Fatty acids are embedded within highly interconnected metabolic and signaling networks, and their effects are unlikely to be pathway specific. For example, genetic variation in *FADS1/2* influences multiple downstream lipid species across both n-3 and n-6 pathways, complicating causal attribution to any single fatty acid class and raising the possibility that observed associations reflect broader lipid regulatory phenotypes rather than isolated deficiencies. Furthermore, current understanding of the immunological effects of PUFAs may be overly simplistic, as recent MR evidence found no convincing support for a broadly pro- or anti-inflammatory role of n-6 or n-3 PUFAs (252). Further research is therefore needed to better elucidate the mechanisms underlying PUFA effects on immune biomarkers. In addition, schizophrenia and bipolar disorder are highly polygenic in nature, with multiple genetic and additionally environmental disease risk factors at play. Therefore, the pathways through which PUFAs may influence psychosis are also likely to be heterogeneous across individuals, encompassing modulation of neuroinflammatory processes, alteration of membrane phospholipid composition and receptor signaling, effects on neurotransmitter systems, promotion of synaptic plasticity, and generation of specialized pro-resolving lipid mediators. Consequently, studies focusing on different clinical populations or disease stages may be targeting distinct underlying pathophysiological processes, further complicating interpretation of findings. This biological complexity is further compounded by the difficulty of isolating dietary effects in clinical trials. PUFA intake is embedded within broader dietary patterns, which are themselves associated with symptom severity, inflammation, socioeconomic status, and cardiometabolic risk. As a result, supplementation studies are challenging to design and interpret, particularly when baseline dietary intake varies widely across populations.

An additional layer of complexity arises from the systemic nature of PUFA biology in psychotic disorders. PUFA supplementation may exert multi-system effects that extend beyond central nervous system function. This is particularly relevant in psychosis and bipolar disorder, where comorbidities are common. For instance, smoking prevalence is substantially elevated in both schizophrenia and bipolar disorder, and n-3 fatty acids have been suggested to confer protective effects against arterial and inflammatory damage associated with long-term tobacco exposure (253). Similarly, individuals with psychotic disorders experience markedly increased rates of cardiometabolic disease, which may partly reflect shared dysregulation of lipid metabolism (254). In this context, the established cardioprotective effects of EPA- and n-6-containing lipid pathways further complicate interpretation, as improvements in peripheral metabolic health may indirectly influence neuropsychiatric outcomes, blurring mechanistic specificity.

Together, these challenges highlight that PUFA research in psychosis and bipolar disorder sits at the intersection of complex lipid biology, heterogeneous clinical phenotypes, and difficult-to-control nutritional and methodological confounds. This complexity may partly explain the inconsistent efficacy signals observed across clinical trials and underscores the need for integrative approaches that consider genomic background such as *FADS* variants, baseline PUFA levels and dietary context, and systemic metabolic health alongside central neurobiological mechanisms. Given the overall efficacy of n-3 supplementation remains uncertain, this has prompted increasing interest in alternative approaches aimed at modulating PUFA-related pathways through pharmacological interventions.

## 12 Therapeutic modulation of PUFA pathways in bipolar disorder and schizophrenia

An alternative perspective that bears consideration is to treat PUFA metabolism itself as the therapeutic target rather than omega-3 supplementation as the intervention. From this viewpoint, pharmacological agents that influence PUFA synthesis, membrane incorporation, downstream lipid mediator production, or related inflammatory pathways may offer alternative routes through which PUFA biology can be leveraged therapeutically. As discussed, increasing attention has focused on the interplay between PUFA metabolism and key psychiatric risk pathways, including glutamatergic and dopaminergic neurotransmission, neuroimmune signaling and synaptic plasticity. Notably, many of these processes are also modulated by antipsychotics, mood stabilizers, and antidepressants (131), suggesting shared biological mechanisms through which nutritional and pharmacological factors may influence psychiatric outcomes.

Intriguingly, several commonly prescribed psychiatric medications have been shown to affect PUFA metabolism. In rats, antipsychotic medications upregulated genes involved in PUFA biosynthesis, with risperidone and paliperidone increasing hepatic *FADS2* mRNA expression and enhancing plasma n-3 and n-6 PUFA biosynthesis (255). Consistent with these findings, a small study of first-episode psychosis patients reported increased erythrocyte PUFA levels following 6 months of antipsychotic treatment, which correlated with improvements in learning and memory (256). However, symptom improvement may have been accompanied by improvements in dietary quality. In addition, other studies have failed to observe changes following sub-chronic treatment with either haloperidol or clozapine in rodents (257). Further studies are thus needed to disentangle the contribution of antipsychotic treatments in PUFA metabolism.

Mood stabilizers frequently used in bipolar disorder, including lithium chloride, valproic acid, and carbamazepine, reduced brain AA turnover, but not DHA turnover, when administered to rats at therapeutically relevant doses (61). Chronic lithium treatment also increased AA levels and the AA ratio in blood and brain tissue of mice maintained on a control diet (168), whereas another study reported increased brain DHA formation alongside elevated AA-derived metabolites following lithium administration (258). Interestingly, a metabolome-wide MR study reported suggestive evidence that lipid metabolites associated with lower bipolar disorder risk, including AA, were also associated with a higher likelihood of favorable lithium response (216) suggesting a potential interaction between PUFA metabolism and lithium mechanisms of action.

Nevertheless, mechanistic evidence indicates that antipsychotic medications influence lipid signaling pathways. *In vitro* studies have shown that common antipsychotics modulate expression of sterol regulatory element-binding protein (*SREBP*) transcription factors in human glial cells, a central regulator of cellular lipogenesis and lipid homeostasis (259) that is itself modulated by PUFA availability (260). Furthermore, clozapine and risperidone enhanced hepatic lipogenesis and cholesterogenesis in rats through induction of SREBP-dependent pathways (261). Therefore, psychiatric medications can influence multiple aspects of PUFA metabolism and lipid signaling, raising the possibility that modulation of these pathways may contribute, at least in part, to their therapeutic effects.

Interest has also emerged in drugs that target lipid metabolism pathways, including the widely prescribed family of drugs statins. Statin use was associated with significantly lower rates of incident mania and bipolar disorder in an analysis of more than one million individuals from a Danish nationwide registry (12) and reduced rates of self-harm and hospitalization in patients with bipolar disorder and schizophrenia in another study (262). In addition, small trials of statins as adjunctive therapy in schizophrenia have shown improvements in positive and negative symptoms over and above routine antipsychotic treatment (263, 264). Both preclinical and clinical data indicate that statins upregulate desaturase (*FADS1*, *FADS2*) and elongase (*ELOVL2*, *ELOVL5*) expression, promoting LC-PUFA biosynthesis (265–267). Consistent with this, statin therapy was associated with a relative decrease in SC-PUFAs LA and ALA, and an increase in AA and DHA proportions of total fatty acids (268). In addition, through inhibition of HMG-CoA reductase, statins reduce the synthesis of downstream isoprenoids, leading to altered signaling pathways that regulate the expression of genes involved in lipid metabolism (266). Statins may also alter PUFA transport and distribution through reductions in circulating very-low-density lipoproteins (VLDL) and low-density lipoproteins (LDL), which carry fatty acids in the bloodstream (269). Furthermore, changes in membrane cholesterol content may influence PUFA incorporation into phospholipids and consequently affect downstream lipid signaling pathways, including eicosanoid production. Together, these findings suggest that statins may modulate PUFA metabolism through multiple mechanisms. As such, repurposing statins to target LC-PUFA biosynthesis in patients and at-risk individuals may represent a promising avenue for future investigation, especially given their benign side effect profile.

Rather than attempting to increase PUFA availability through dietary supplementation or enhanced biosynthesis, an alternative strategy is to pharmacologically target downstream PUFA-derived signaling pathways. Commonly used non-steroidal anti-inflammatory drugs (NSAIDs), such as ibuprofen and aspirin, act by blocking COX-mediated conversion of AA-derived PUFAs into prostaglandins, thereby shifting downstream lipid signaling away from pro-inflammatory eicosanoid production. Selective COX-2 inhibitors (e.g., celecoxib) have also been trialed as adjunctive treatments in schizophrenia. In this context, a meta-analysis by Zheng et al. (270) concluded that adjunctive celecoxib may be an efficacious and safe treatment for improving psychotic symptoms, particularly in first-episode schizophrenia. More broadly, a meta-analysis by Nitta et al. (271) found that NSAIDs may confer benefits on positive symptoms when used as adjuncts, although effects were modest. In addition, a recent retrospective analysis of clinical records reported that low-dose aspirin co-treatment was associated with improved clinical outcomes across bipolar disorder, schizophrenia, and schizoaffective disorder (272). Taken together, these findings suggest that pharmacological modulation of COX/LOX pathways may be therapeutically relevant in psychosis, underscoring the role of downstream PUFA-derived signaling and neuroinflammatory mechanisms. Whether such therapies might act synergistically with n-3 PUFA supplementation remains unexplored.

To conclude, these findings suggest that PUFA biology may be therapeutically relevant even when direct n-3 supplementation yields inconsistent clinical outcomes. Nonetheless, the complexity of PUFA metabolism and its interactions with genetic, dietary, inflammatory, and pharmacological factors still present substantial challenges for clinical translation. This highlights the need for further research into both pharmacological agents that modulate PUFA pathways, particularly commonly used drugs that may influence endogenous PUFA synthesis or selectively reroute bioactive lipid mediators. While such approaches remain underexplored, they represent a promising direction for future therapeutic development.

## 13 Conclusion and future directions

In conclusion, converging lines of evidence support a biologically plausible role for n-3 PUFAs in the etiology and pathophysiology of psychotic disorders, with particularly strong evidence for bipolar disorder. Mechanistic studies demonstrate that n-3 PUFAs are key regulators of neurodevelopment, neuroinflammation, and neuronal signaling, while early-life PUFA deficiency in preclinical animal models can produce behavioral and neurobiological alterations relevant to these psychiatric conditions. Human observational studies consistently report abnormalities in PUFA status in schizophrenia and bipolar disorder, particularly low peripheral LC-PUFA levels and imbalanced n-3:n-6 ratios reflecting an overabundance of n-6 relative to n-3. Genomic evidence further strengthens the case for a causal role of PUFA metabolism, particularly through variants within the *FADS* gene cluster that reduce desaturase activity. These variants have been associated with disease risk, with the strongest evidence emerging in bipolar disorder, and suggest that more efficient LC-PUFA conversion may play a protective role. Impaired conversion of n-3 and n-6 SC-PUFA precursors may result in accumulation of precursor fatty acids and/or relative deficiency of LC-PUFAs, highlighting altered PUFA metabolism as a potential disease mechanism in both bipolar disorder and schizophrenia. This likely has widespread downstream consequences across the many biological processes mediated by PUFAs. However, despite this substantial body of mechanistic, epidemiological, and genetic evidence, clinical intervention trials have yielded inconsistent results. This likely reflects a combination of methodological limitations, including heterogeneity in study populations, variation in PUFA formulations and dosing regimens, inadequate consideration of baseline PUFA status and failure to account for genetic factors affecting PUFA metabolism. Nevertheless, encouraging preliminary findings in UHR and FEP populations suggest that n-3 PUFA supplementation may have therapeutic potential when administered early in the disease course.

Several key questions remain unresolved. It is still unclear whether low dietary intake of n-3 LC-PUFAs, or an elevated n-6:n-3 ratio, may compound genetically mediated reductions in endogenous LC-PUFA synthesis in individuals carrying variants in desaturase genes such as *FADS2*, thereby exacerbating functional PUFA deficiency and increasing susceptibility to psychiatric and other related morbidities. In addition, mechanistic preclinical animal models, and in-depth human longitudinal studies, are needed to determine the importance of developmental timing of PUFA exposure. Specifically, it is unclear whether adequate PUFA availability is necessary during early neurodevelopment for shaping long-term psychiatric risk, or whether targeted supplementation later in development can still meaningfully modify outcomes by influencing ongoing disease processes such as adolescent synaptic pruning of late-maturing brain regions, which occur closer to typical disease onset. In parallel, it remains to be determined whether interventions targeting PUFA pathways, either through dietary supplementation or through pharmacological modulation of downstream lipid signaling cascades (e.g., statins or NSAIDs), are most effective in individuals stratified by genetic background or baseline PUFA status, particularly those with *FADS2* risk alleles or low circulating LC-PUFA levels. Finally, there is a need for mechanistic work to clarify how *FADS2* variation influences cellular and neurodevelopmental processes, including membrane composition, synaptic function, and neuroimmune signaling, and how these changes may contribute to altered risk trajectories for psychiatric disorders.

Future research should move beyond descriptive PUFA profiling toward mechanistic investigations of lipid metabolism and signaling pathways. While current genomic and epidemiological evidence increasingly implicates impaired PUFA biosynthesis as a potential upstream risk mechanism, the cellular and molecular pathways linking altered PUFA metabolism to psychosis remain poorly understood. Given the broad involvement of PUFAs in neurodevelopment, neurotransmission, myelination, stress responsivity, and immune regulation, further work is needed to elucidate how FADS-mediated variation in PUFA metabolism influences these interconnected risk pathways. Emerging proteomic and lipidomic approaches offer an opportunity to address the fragmented nature of the current literature by examining PUFAs within broader lipid networks and elucidating their roles as bioactive signaling molecules in psychosis pathophysiology (60, 206, 273, 274). Furthermore, baseline PUFA status and *FADS* genotypes should be routinely assessed in clinical trials to facilitate patient stratification, identify subgroups most likely to benefit from intervention, and improve study sensitivity, precision, and interpretation (33). Such approaches may ultimately support the development of more targeted and personalized nutritional and metabolic interventions for psychotic disorders.
